# Toxin import through the antibiotic efflux channel TolC

**DOI:** 10.1038/s41467-021-24930-y

**Published:** 2021-07-30

**Authors:** Nicholas G. Housden, Melissa N. Webby, Edward D. Lowe, Tarick J. El-Baba, Renata Kaminska, Christina Redfield, Carol V. Robinson, Colin Kleanthous

**Affiliations:** 1 Department of Biochemistry, University of Oxford, Oxford, UK; 2 Physical and Theoretical Chemistry Laboratory, Department of Chemistry, University of Oxford, Oxford, UK

**Keywords:** Membrane proteins, Antibiotics, Bacterial structural biology, Bacterial toxins

## Abstract

Bacteria often secrete diffusible protein toxins (bacteriocins) to kill bystander cells during interbacterial competition. Here, we use biochemical, biophysical and structural analyses to show how a bacteriocin exploits TolC, a major outer-membrane antibiotic efflux channel in Gram-negative bacteria, to transport itself across the outer membrane of target cells. Klebicin C (KlebC), a rRNase toxin produced by *Klebsiella pneumoniae*, binds TolC of a related species (*K. quasipneumoniae*) with high affinity through an N-terminal, elongated helical hairpin domain common amongst bacteriocins. The KlebC helical hairpin opens like a switchblade to bind TolC. A cryo-EM structure of this partially translocated state, at 3.1 Å resolution, reveals that KlebC associates along the length of the TolC channel. Thereafter, the unstructured N-terminus of KlebC protrudes beyond the TolC iris, presenting a TonB-box sequence to the periplasm. Association with proton-motive force-linked TonB in the inner membrane drives toxin import through the channel. Finally, we demonstrate that KlebC binding to TolC blocks drug efflux from bacteria. Our results indicate that TolC, in addition to its known role in antibiotic export, can function as a protein import channel for bacteriocins.

## Abstract (web summary)

Bacteria can secrete diffusible protein toxins that kill competing bacteria. Here, the authors use biochemical, biophysical and structural analyses to show how one of these toxins exploits TolC (a major antibiotic efflux channel) to transport itself across the outer membrane of target cells.

## Introduction

The outer membrane (OM) of Gram-negative bacteria is comprised of a phospholipid inner leaflet and an outer leaflet of lipopolysaccharides cross-linked by chelated divalent cations. This asymmetric membrane forms a robust structure that stabilises the cell envelope and secures bacterial survival in challenging environments. The OM is also a virulence factor in pathogens and, through its exclusion of both hydrophobic and hydrophilic compounds, a major component of antibiotic resistance in Gram-negative bacteria^1^. Small (<700 Da) antibiotics can nevertheless cross the OM through porins such as OmpF and OmpC that pepper the bacterial surface^2^. To counter the effects of antibiotics, bacteria adapt by reducing the permeability of the OM, inactivating or hydrolysing the antibiotic or actively expelling the drug from the cell. TolC is one of the most prevalent drug efflux channels in bacteria and is frequently associated with multidrug resistance^3^. TolC also forms the exit tunnel for type I secretion of hemolysin, a pore-forming toxin that lyses red blood cells^4^.

Bacteriocins are diffusible peptide or protein antimicrobial toxins deployed by Gram-positive and Gram-negative bacteria to kill their neighbours in the unremitting warfare between and within bacterial populations^5^. Protein bacteriocins (herein referred to simply as bacteriocins) are weaponry often used by *Enterobacteriaceae* and *Pseudomonaceae* that are living in ecological niches as diverse as soil, plants, animals and humans^6^. Bacteriocin genes are often associated with virulence factors, suggesting they help pathogens become established during pathogenesis^7^. Bacteriocins tend to be species-selective, killing only closely related bacteria due to the specific protein–protein interactions involved in binding surface receptors and translocating into the cell. Their import is driven by the proton-motive force (PMF) in conjunction with either the Tol (group A bacteriocins) or the Ton (group B bacteriocins) systems^8^. The physiological roles of the Tol (also known as Tol-Pal) and Ton systems are to stabilise the OM during cell division^9^ and transport nutrients through TonB-dependent transporters (TBDTs)^10^, respectively. These functions are parasitized by bacteriocins to catalyse their transfer across the OM. Following internalisation, cell death ensues through the delivery of a cytotoxic domain located at the C-terminus of the toxin that degrades DNA, rRNA or tRNA in the cytoplasm, depolarises the inner membrane or cleaves peptidoglycan precursors in the periplasm. Bacteriocin-producing bacteria are immune to the specific action of their toxin by virtue of a small immunity protein that binds and inactivates the toxin and which, in the case of nucleases, is jettisoned during import^11,12^.

Much is known about the associations of bacteriocins with OM receptors, translocator proteins and components of the Tol or Ton systems, but not how they cross the OM since no bacteriocin has yet been trapped and structurally characterised within its OM translocon. Nevertheless, the routes taken by these toxins to cross the OM are beginning to be mapped out. For example, photoactivatable cross-linking studies have shown that the group B bacteriocin pyocin S2 (PyoS2), a nuclease deployed by *Pseudomonas aeruginosa*, translocates directly through a TBDT^13^. Following binding to the common polysaccharide antigen^14^, PyoS2 associates with the pyoverdine transporter, FpvAI. Import through FpvAI is a two-step process; both steps are driven by the PMF, transduced to the OM by TonB in conjunction with its inner membrane stator protein partners, ExbB and ExbD. In the first step, the plug domain that normally occludes the central channel of FpvAI is partially opened by TonB. This occurs by TonB mechanically deforming the plug through its association with a TonB-box epitope at the N-terminus of FpvAI in the periplasm. In the second step, PyoS2 delivers its own TonB box, in the form of a disordered linear epitope, through the now-open FpvAI channel that engages TonB and enables the toxin to be pulled unfolded through the transporter.

In this work, we define the import pathway for a family of group B bacteriocins found amongst the *Enterobacteriaceae*. Focusing on the 60-kDa rRNase bacteriocin klebicin C (KlebC) from *Klebsiella pneumoniae*, we show how KlebC exploits the antibiotic efflux channel TolC to transport itself across the OM. As part of this work, we structurally characterise a trapped complex formed between KlebC and the TolC translocation channel.

## Results

### Identification of the KlebC outer-membrane receptor

One-third of all *K. pneumoniae* strains recently surveyed encode one or more nuclease bacteriocins which, despite low-sequence identity, have similar domain organisation to that seen in other bacterial species, principally *Escherichia coli, Salmonella enterica, Serratia marcescens, Shigella sonnei* and *Enterobacter cloacae*^7^. Nuclease bacteriocins in these species typically have disordered N-termini followed by one or more structured domains involved in import and a nuclease domain at the C-terminus. This work focuses on KlebC, a little-studied rRNase bacteriocin. rRNase bacteriocins kill target cells by cleaving a single phosphodiester bond within the ribosomal A-site of the 70S ribosome. While the mechanism by which bacteriocin rRNase domains inhibit protein synthesis is well-understood^15^, it is largely unknown how such domains cross the OM. Hence, we set out to define the KlebC import mechanism by first identifying its OM receptor and/or translocator by affinity purification, using an approach similar to that used to define the OM translocon for the *E. coli*-specific bacteriocin, colicin E9^16^. Initial attempts to express KlebC as a heterodimeric complex with its immunity protein and a C-terminal histidine tag—an approach used previously for nuclease colicins^17^—was hampered by toxicity. This was circumvented through the construction of a KlebC-E9 DNase hybrid, in which the highly toxic rRNase domain of KlebC was replaced by the DNase domain of colicin E9 (ColE9) (Supplementary Fig. 1a). This hybrid protein was readily expressed and purified as a heterodimeric complex via a C-terminal His tag on its immunity protein Im9. The isolated hybrid protein killed *Klebsiella pneumoniae, Klebsiella varricola and Klebsiella quasipneumoniae*, in addition to possessing significant activity against *E. coli* (Supplementary Fig. 1b). These experiments demonstrated that the remaining KlebC domains harbour all the information necessary to transport the nuclease into bacteria. Importantly, the cytotoxicity of KlebC-E9 DNase hybrid (hereafter referred to as KlebC) against *E. coli* allowed us to test select stains from the Keio *E. coli* knockout collection^18^ as a means of identifying important components of the import pathway. Hence, KlebC was shown to be a TonB-dependent, group B bacteriocin since the *tonB* knockout was completely resistant to its action whereas the *tolA* deletion remained sensitive (Supplementary Fig. 1b).

We performed a series of pull-down assays to identify KlebC OM binding partners. Purified KlebC was added to cultures of *K. quasipneumoniae*, followed by lysis, outer-membrane purification, detergent extraction, and nickel affinity chromatography. Imidazole eluate from the nickel affinity column was analysed by SDS-PAGE, revealing three prominent bands, which were identified by peptide mass fingerprinting (Supplementary Fig. 1c). The 70 kDa band was found to be the bait protein KlebC, and the 50 kDa band was identified as the OM efflux channel TolC (45 peptides, 92.8% sequence coverage). The 34 kDa band contained peptides from degraded bait protein in addition to 7 peptides from the OM protein OmpA (22.8% sequence coverage). We further investigated the roles of TolC and OmpA in KlebC uptake using BW25113 *ompA* and BW25113 *tolC* knockout strains (Supplementary Fig. 1d). The former showed no impact on KlebC toxicity suggesting it to be a contaminant due to its high natural abundance, whilst the latter was resistant. Although TolC was critical for KlebC toxicity its inner membrane partners AcrA and AcrB, which energise drug efflux from bacteria^19^, were not involved in KlebC function (Supplementary Fig. 1d). While several colicins (E1, 5 and 10) are known to kill *E. coli* in a TolC-dependent manner^20–22^ and share sequence identity (55%) with the N-terminal domain of KlebC, it remains unclear what role TolC plays in the import mechanism. Indeed, in the case of ColE1, it is thought that TolC simply constitutes a docking site prior to OM translocation^23^. We conclude that KlebC is a TolC-dependent, group B bacteriocin that kills *K. pneumoniae* and closely related species. Furthermore, an N-terminal domain implicated in KlebC uptake via TolC by an ill-defined mechanism is conserved amongst other TolC-utilising bacteriocins targeting different species (Supplementary Fig. 1e). Finally, we identified a TonB-box motif in KlebC (M^16^VSLG^20^) the mutation of which abolished toxicity (Supplementary Fig. 1f). This result is consistent with KlebC contacting TonB during import, which is a requirement for all group B bacteriocins^5^.

### Structure and binding mechanism of the TolC-binding domain of KlebC

**Fig. 1:**
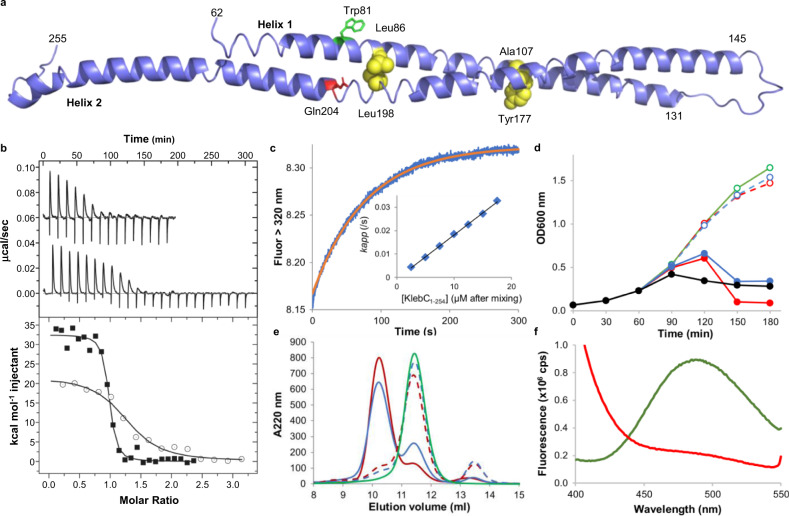
Structure of the KlebC–TolC-binding domain and its binding to KqTolC. **a** Cartoon representation of the KlebC_51-254_ TolC-binding domain crystal structure. Leu86, Ala107, Tyr177 and Leu198, mutated to form disulphide bonds are shown as yellow spheres. Side chains of FRET pair Trp81 (green sticks) and Gln204 (red sticks), which was mutated to Cys, are also shown. **b** ITC titration of 84 µM KlebC_1-254_ into 7.6 µM *Kq*TolC (filled squares) and 89 µM KlebC_51-254_ into 5.9 µM *Kq*TolC (offset by 0.06 µcal s^−1^ in top panel and open circles in bottom panel). Data were fitted to a one set of sites fit to give *K*_d_ = 35 ± 7 nM, *N* = 0.90 ± 0.03 binding sites per TolC trimer and ΔH 30.6 ± 2.6 kcal mol^−1^ and *K*_d_ = 368 ± 16 nM, *N* = 1.34 ± 0.12 binding sites per TolC trimer and Δ*H* 20.8 ± 1.0 kcal mol^−1^ for KlebC_1-254_ and KlebC_51-254_, respectively. Typical traces are shown, values are averages of duplicate experiments. **c** Pre-equilibrium fluorescence increase in tryptophan emission upon complex formation between 0.5 µM *Kq*TolC and 7.5 µM KlebC_1-254_. Single exponential fit to the data to determine *k*_app_ is shown in red. Inset, Dependence of *k*_app_ on KlebC_1-254_ concentration. Data are averages of duplicate experiments fitted to a straight line, the gradient of which gives the association rate constant, 1.9 ± 0.1 × 10^3^ M^−1^ s^−1^. **d** Liquid growth curves of *K. quasipneumoniae* Qmp M1-977, with the addition of KlebC-E9 DNase (black), A107C, Y177C KlebC-E9 DNase (reduced, solid red; oxidised, dashed red), L86C, L198C KlebC-E9 DNase (reduced, solid blue; oxidised, dashed blue), or no KlebC-E9 DNase (green), added after 60 min. Data shown are from one representative experiment out of three biological repeats. **e** In total, 200 µl *Kq*TolC (green), *Kq*TolC + L86C, L198C KlebC_1-254_ (reduced, solid blue; oxidised, dashed blue), and *Kq*TolC + A107C, Y177C KlebC_1-254_ (reduced, solid red; oxidised, dashed red) were loaded onto a 10/300 S200 column. **f** Fluorescence emission of 1 µM Q204C^AEDANS^ KlebC_1-254_ in the absence (green) and presence (red) of 1 µM *Kq*TolC. *λ*_Ex_ = 280 nm, slit widths = 2 nm.

We next set out to delineate the TolC-binding region of KlebC and determine its structure; no structure has yet been reported for any TolC-binding domain. Secondary structure predictions for KlebC suggest the first 53 residues are disordered and are followed by a region of 218 residues with high α-helical propensity, with a break in helicity between residues 251 and 255. This region also encompassed sequence related to other TolC-dependent bacteriocins (Supplementary Fig. 1e). Three constructs encoding residues 1–271, 1–254 and 51–254 were cloned with C-terminal His_6_-tags and their ability to bind *K. quasipneumoniae* TolC was assessed using analytical gel-filtration chromatography. TolC eluted at a volume of 12.0 ml, whilst the elution peaks of TolC in the presence of KlebC_1-271_, KlebC_1-254_ and KlebC_51-254_ shifted to 10.6, 10.8, and 11 ml, respectively, consistent with complex formation (Supplementary Fig. 2). Crystallisation screens were focused solely on KlebC_51-254_ since this construct retained TolC-binding activity (similar to KlebC_1-271_) whilst removing the putative disordered region at the N-terminus. Crystals of selenomethionine-labelled KlebC_51-254_ were obtained in the P3_2_21 space group. The resulting structure, solved by single-wavelength anomalous diffraction to a resolution of 1.9 Å, revealed a helical hairpin spanning 150 Å (Fig. 1a). Modelled density spanned Lys62 to Leu255, with residues 64–131 forming helix 1 of the hairpin and helix 2 containing residues 145–248. The lack of density for residues 51–61 suggest that the disorder predicted for the first 50 residues may extend further into the molecule. We explored this possibility with heteronuclear NMR experiments and found that indeed the first 60 residues of KlebC are disordered in solution (Supplementary Fig. 3). Residues 62–73 in the structured hairpin domain are weakly helical, as evidenced by peptide bond characteristics atypical of standard α-helix.

We next investigated how the helical-hairpin domain of KlebC bound TolC using different biophysical approaches and domain constructs with or without the disordered region at the N-terminus. Isothermal titration calorimetry (ITC) demonstrated that a single molecule of KlebC_1-254_ bound the TolC trimer (*N* = 0.90 ± 0.03) with a *K*_d_ of 35 ± 7 nM through an entropically driven, endothermic interaction (Fig. 1b). Truncated KlebC_51-254_ bound TolC with an affinity approximately tenfold weaker (*K*_d_ = 368 ± 16 nM, *N* = 1.34 ± 0.12) (Fig. 1b), demonstrating that although disordered, the N-terminal region of KlebC plays a role in the TolC interaction. The binding kinetics of KlebC for TolC were also investigated, using intrinsic fluorescence and capitalising on the 12% enhancement in tryptophan emission fluorescence (*λ*_ex_, 295 nm/*λ*_em_, 340 nm) for the complex. Pre-equilibrium fluorescence measurements revealed binding to be very slow, suggestive of a single-step process occurring with a bimolecular association rate constant (*k*_on_) of 1.9 ± 0.1 × 10^3^ M^−1^ s^−1^ (Fig. 1c). Having established the *K*_d_ for the complex by ITC, the dissociation rate constant (*k*_off_), which was too slow to be measured (insert, Fig. 1c), was estimated to be ~5 × 10^−5^ s^−1^, assuming the kinetically determined *K*_d_ = *k*_off_/*k*_on_. Typical protein–protein interactions have association rate constants >10^5^ M^−1^ s^−1^. Ultra-slow association, such as that observed for the KlebC_1-254_–TolC complex, is often indicative of gross conformational changes accompanying complex formation^24^.

We hypothesised that the kinetics of association and thermodynamic signature of KlebC–TolC complex formation were the results of the KlebC helical-hairpin unfurling. We tested this hypothesis in two ways. The first approach involved introducing disulphide bond crosslinks across the helical hairpin of KlebC which could prevent the hairpin from opening. Using disulphide-by-design^25^, two interhelical disulphide bonds were engineered (L86C, L198C and A107C, Y177C; Fig. 1a) to lock the helical hairpin in the conformation observed in the crystal structure. The introduction of either of these disulphide bonds into KlebC resulted in a toxin active in its reduced form, but inactive in the oxidised state (Fig. 1d). To determine if the disulphides within KlebC had inactivated the toxin by preventing translocation following receptor binding, or prevented receptor binding itself, the disulphides were engineered into KlebC_1-254_ and complex formation with TolC investigated by analytical gel-filtration chromatography. In their reduced forms, both L86C, L198C KlebC_1-254_ and A107C, Y177C KlebC_1-254_ bound TolC, but in their oxidised states neither protein could bind TolC (Fig. 1e). These experiments indicate that TolC binding requires the helical hairpin to undergo a gross conformational change from that observed in the crystal structure.

We applied a second approach using a FRET-based assay to determine whether the helical hairpin of KlebC_1-254_ unfurled on association with TolC. The TolC-binding domain of KlebC contains a single tryptophan (W81) located within helix 1. A cysteine was introduced into helix 2 of KlebC_1-254_ (Q204C), in close proximity to W81 allowing for labelling with 5-((((2-iodoacetyl)amino)ethyl)amino)naphthalene-1-sulfonic acid (IAEDANS) and the creation of a Trp-AEDANS FRET pair that was used to monitor the conformation of the hairpin. Using an excitation wavelength of 280 nm, and in the absence of TolC, Q204C^AEDANS^ KlebC_1-254_ gave a Trp-to-AEDANS FRET signal at 490 nm, which upon addition of equimolar TolC trimer decreased by 77% (Fig. 1f), which is consistent with the hairpin unfurling during complex formation. Loss of the Trp-to-AEDANS FRET signal upon TolC binding was further investigated under pre-equilibrium conditions. Experiments performed under pseudo-first-order conditions, with either Q204C^AEDANS^ KlebC_1-254_ or TolC in excess resulted in a single-phase fluorescence decrease (Supplementary Fig. 4), which was fitted to a single exponential equation to give *k*_app_. From the variation in *k*_app_ with protein concentration, association rate constants of 6.7 ± 0.2 × 10^3^ M^−1^ s^−1^ and 2.9 ± 0.1 × 10^3^ M^−1^ s^−1^ were calculated, with TolC and Q204C^AEDANS^ KlebC_1-254_ in excess, respectively. Notwithstanding the slow association rate constant, the requirement of the TolC-binding domain of KlebC to undergo a significant conformational rearrangement prior to binding does not appear to be rate-limiting. This interpretation is suggested by the observation that with either component in excess a single-phase fluorescence change is observed and that the observed association rate constant in the presence of excess TolC was only 2.3-fold faster than when Q204C^AEDANS^ KlebC_1-254_ was in excess. Hence, our kinetic data suggest that a significant fraction of KlebC_1-254_ in solution is in a binding-competent form (~40%). Taken together, these data demonstrate that the N-terminal helical-hairpin domain of KlebC must unfurl in order to bind TolC, that unfurling is not rate-limiting for TolC binding and that this conformational change is a requirement for TolC-mediated killing of bacterial cells by KlebC.

### Structure of *Klebsiella quasipneumoniae* TolC and its complex with KlebC_1-254_

*K. quasipneumoniae* Qmp M1-977 is a KlebC susceptible strain in which TolC (*Kq*TolC) shares 83% sequence identity with *E. coli* K-12 TolC. *Kq*TolC was expressed and purified for single-particle cryo-EM studies to resolve the structure of the *Kq*TolC-KlebC_1-254_ complex. *Kq*TolC-KlebC_1-254_ was mixed in β-OG and transferred into amphipol A8-35 after which image processing resulted in the structure of *K. quasipneumoniae* TolC at a resolution of 3.4 Å (Supplementary Figs. 5–7). However, this structure only resolved *Kq*TolC, which nevertheless served as a useful control. *Kq*TolC is a 150-kDa homotrimer comprised of 50-kDa subunits, each contributing four β-strands to form a single 12-stranded β-barrel across the outer membrane. The remainder of each subunit adopts a helical structure forming an elongated channel. Whilst the β-barrel in the membrane is exposed to the extracellular environment the α-helical segment of the protein forms an iris in the periplasm. The structure of *Kq*TolC differs from that of *E. coli*^26^ through a six-residue deletion in an extracellular loop extending from the β-barrel. The position of extracellular loops within the structure defines the accessible size of the β-barrel, creating a more open configuration in *Kq*TolC relative to *E. coli* TolC (Supplementary Fig. 7). Although some local distortions are observed at the iris where helices meet, none significantly alter the effective diameter of the iris (4–5 Å at its narrowest point).

**Fig. 2:**
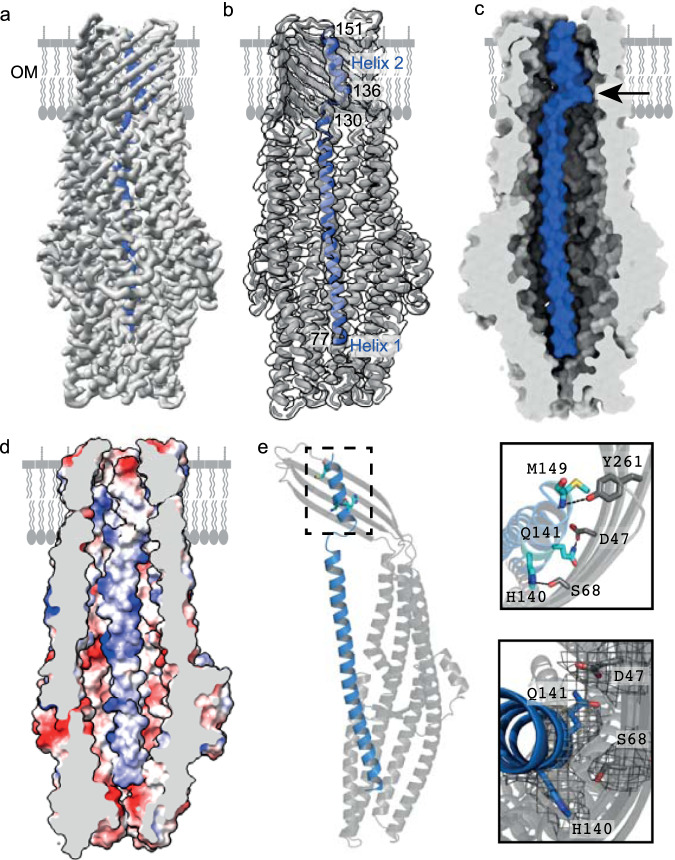
KlebC1-254 binds within the lumen of the KqTolC trimer. **a** Single-particle cryo-EM map of the *Kq*TolC-KlebC_1-254_ complex with KlebC_1-254_ density (blue) resolved within *Kq*TolC (grey). The map resolution extends from 3 to 4 Å. **b** Cut through of *Kq*TolC-KlebC_1-254_ complex map in panel a with the fitted model, shows KlebC_1-254_ density (blue) within the *Kq*TolC channel (grey). **c** Surface representation of the *Kq*TolC-KlebC_1-254_ complex highlighting fit of KlebC_1-254_ in the lumen of *Kq*TolC. The shape of the *Kq*TolC channel combined with interactions between the helices of KlebC_1-254_ and *Kq*TolC, result in KlebC_1-254_ forming a kink (denoted by the arrow) in order to pass through the pore unobstructed. **d** Model of the electrostatic surface for the *Kq*TolC-KlebC_1-254_ complex (the protein sequence for helix 1 is depicted in place of polyalanine used to solve the structure), highlighting the charge matching between the two proteins. Electrostatics generated in chimera. **e** *Kq*TolC chain C shown as a cartoon (grey) interacts with KlebC_1-254_ (blue cartoon) helix 2. Insets show zoomed view of key interactions between *Kq*TolC chain C (grey sticks) and KlebC_1-254_ (cyan sticks), which are localised predominantly to the N-terminal end of helix 2. Cryo-EM map density for modelled residues is shown as inset bottom right.

The cryo-EM structure of the *Kq*TolC-KlebC_1-254_ complex was eventually obtained following stabilisation of the complex by formaldehyde cross-linking prior to detergent exchange into amphipol A8-35 (Fig. 2). Initial processing of the micrographs produced a map with pseudo C3 symmetry, although no symmetry was applied during processing (Supplementary Fig. 5). In this map, three copies of KlebC helix 2 were resolved within the TolC β-barrel, contrasting biophysical data that suggest one KlebC is bound per TolC trimer. To improve the map quality and further resolve KlebC density to get further insight into the binding mode, the amphipol shell surrounding the β-barrel domain of TolC was subtracted and C3 symmetry applied, increasing map resolution from 3.4 to 2.8 Å. Application of symmetry during processing then allowed for symmetry expansion of the dataset and subtraction of particles to give a single monomer, after which 3D sorting of particles revealed several classes where KlebC density was weakly apparent. A single class was further processed (see “Methods” and Supplementary Fig. 5) to produce the final 3.1 Å map of the TolC trimer with both helix 1 and 2 of KlebC resolved (Fig. 2a–c). In the final model, density corresponding to helix 1 and the N-terminal end of helix 2 of a single KlebC molecule span 109 Å of the TolC lumen; the N-terminal end of helix 1 is located near the periplasmic iris of TolC while helix 2 resides near the top within the outer-membrane β-barrel. The conformation of KlebC observed within the complex demonstrates unequivocally that the KlebC hairpin unfurls, forming a distorted kinked structure in which helix 1 is positioned against the α-helical region of TolC, and residues 137–151 form helix 2 within the transmembrane β-barrel of TolC (Fig. 2a–c).

In the crystal structure of the KlebC_51-254_ isolated helical hairpin, residues Asp137 to Gln144 comprise the linker sequence between helix 1 and helix 2. By contrast, in the complex with *Kq*TolC this region forms the N-terminal end of helix 2. This change in structure is accommodated by hydrogen bonding interactions between KlebC and TolC. The TolC channel is highly electronegative while the region of KlebC that binds TolC is basic (*pI*, 10). The modelled structure of the complex shows charge complementarity plays an important role in stabilising the complex since several localised regions of positive and negative charge are matched (Fig. 2d). The N-terminus of KlebC helix 1 is oriented such that it is proximal to both chain A and C of the TolC trimer towards the iris, whilst the C-terminus of helix 1 can solely interact with chain A where the helices of TolC meet the β-barrel strands at the inner leaflet of the OM. In contrast, helix 2 of KlebC packs against the β-barrel region of chain C with His140 and Gln141, from within interhelical linker, now forming hydrogen bonds to Ser68 and Asp47 of TolC, respectively (Fig. 2e). Despite residing within the TolC lumen, several regions of KlebC do not come into contact with the channel due to the restricted flexibility of KlebC helix 1. The unfurling of the KlebC_1-254_ helical hairpin required to bind TolC results in the loss of 1994 Å^2^ buried accessible surface area between the two helices. This is compensated for in the complex with TolC by the burial of 2384 Å^2^, predominantly through interactions with subunits A and C of TolC (1170 Å^2^ and 1120 Å^2^, respectively).

In summary, we have structurally resolved a partially translocated state of KlebC, trapped within its receptor/translocator, TolC. The 150-Å long, N-terminal helical-hairpin domain of KlebC unfurls entirely in order to bind the lumen of TolC thereby positioning its N-terminus deep within the periplasm. The resulting complex is stabilised by complementary electrostatic interactions and hydrogen bonds and spans much of the periplasmic helical region of TolC as well as its transmembrane β-barrel domain.

### KlebC_1-254_ projects beyond the iris of *Kq*TolC

In the *Kq*TolC-KlebC_1-254_ complex, the density for helix 1 of KlebC coincides with the tapering of the TolC channel into the iris (Fig. 2c). This suggests that in order for KlebC_1-254_ to adopt its TolC-bound conformation the N-terminal end of helix 1 has to unfold. Helical unfolding would be consistent with the entropically driven binding observed by ITC (Fig. 1b). Two possibilities might account for the absence of N-terminal residues of KlebC from the structure of the complex; either they reside unstructured within the lumen of TolC or they protrude beyond the iris of TolC, which would require local structural perturbations, not resolved in the current structure, to allow an unstructured polypeptide to fit through. We can discount the possibility that these sequences pass between the periplasmic helices of the TolC channel since the positions of the helices are essentially identical in the KlebC-bound and unliganded forms of *Kq*TolC (Supplementary Fig. 7).

**Fig. 3:**
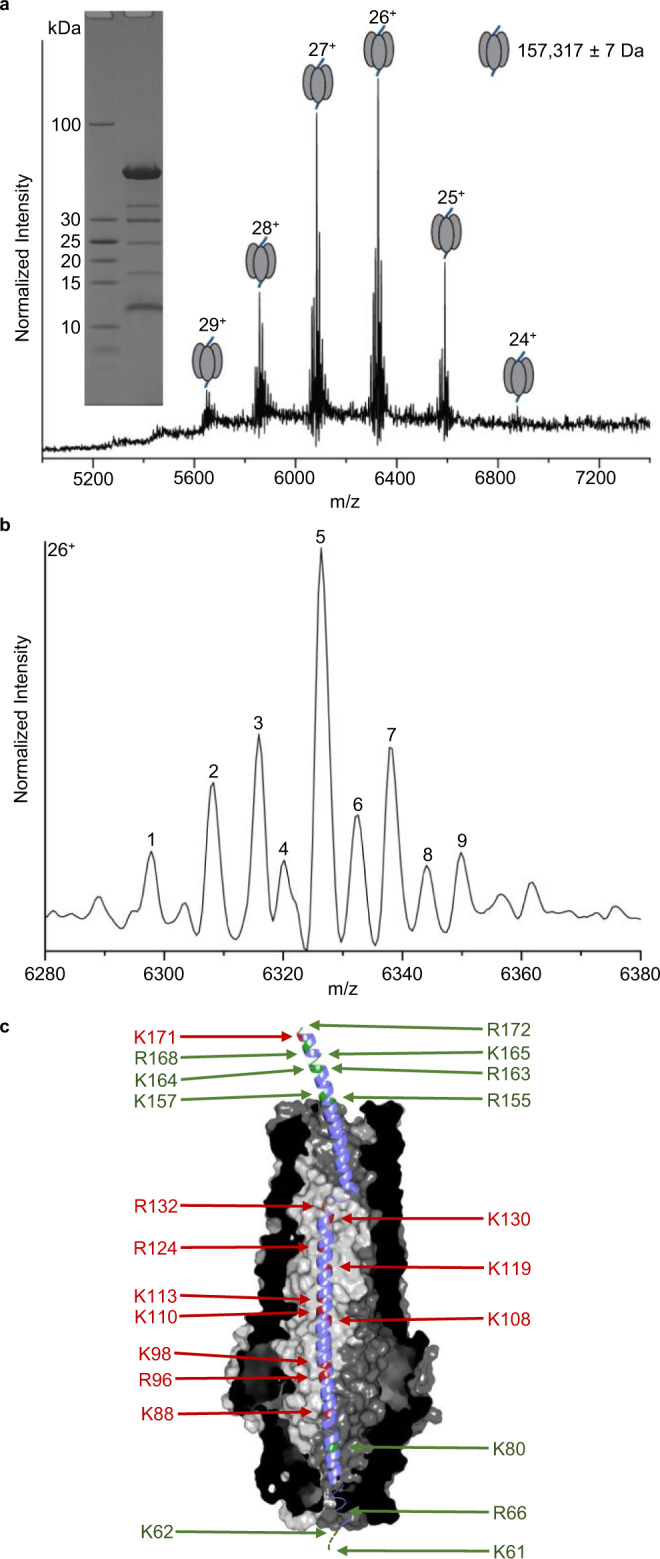
Mapping the regions of KlebC1-254 protected from trypsin by KqTolC. **a** Native-state ESI-MS spectrum of the trypsin-digested *Kq*TolC-KlebC_1-254_ complex. Representative spectra shown from *n* = 3 technical replicates. Inset, 10–20% SDS-PAGE analysis of trypsin-digested *Kq*TolC-KlebC_1-254_ complex after purification by gel-filtration chromatography. **b** Detailed view of the 26^+^ charge state showing heterogeneity of the sample. Numbered peaks are assigned in Supplementary Table 3. **c** Cartoon representation of KlebC_67-163_ bound within the lumen of *Kq*TolC. The figure was constructed by superposing residues 63–81 and residues 145–172 of the KlebC_51-254_ crystal structure onto the KlebC_77-151_ seen within the TolC lumen of the cryo-EM structure. Cleaved trypsin sites are highlighted in green whilst uncut sites are shown in red.

Protease protection assays using trypsin were performed to ascertain how much of the KlebC_1-254_ sequence was solvent accessible in the *Kq*TolC-KlebC_1-254_ complex. *Kq*TolC alone was largely resistant to trypsin, with denaturing mass spectrometry (MS) showing only 13 residues in addition to the His tag cleaved from its C-terminus, whilst KlebC_1-254_ was completely degraded within 5 min by trypsin (Supplementary Fig. 8). Following 120 min of trypsin digestion, the *Kq*TolC-KlebC_1-254_ complex was purified by gel-filtration chromatography and the eluate analysed by both native-state MS and peptide mass fingerprinting of bound (protected) KlebC (Fig. 3). The main species identified by native-state MS was the TolC trimer, truncated at its C-terminus, in complex with a peptide of 10,598 Da, which corresponds to KlebC_67-163_. However, there was considerable heterogeneity in the KlebC peptide fragments identified; starting at residue 63/67/81 and ending at 155/157/163/164/165/168/172, hence results from native-state MS were validated by proteomics. SDS-PAGE analysis of the purified trypsin-digested *Kq*TolC-KlebC_1-254_ complex (Fig. 3a, inset) showed five prominent bands in addition to TolC. Further in-gel trypsin digestion of these bands combined with LC-MS showed peptides >15 kDa were derived from TolC while those ~10 kDa belonged to KlebC. Within the 10-kDa KlebC fragment, three peptides, spanning KlebC_67-80_, KlebC_81-88_ and KlebC_114-124_, were dominant along with several lower abundance peptides (Supplementary Table 4). Combined, these data demonstrate that ~13 cleavage sites within KlebC_1-254_ are protected from trypsin when bound to TolC. These sites span residues 67–163, confirming the identity of the proteolysed complex observed by native-state MS (Fig. 3). Of these, residues 77–151 are resolved in the cryo-EM structure of the complex. Heterogeneity in the sites of KlebC cleavage likely reflects dynamics within the complex, with observed cleavage at K80 requiring deviation from the state observed in the cryo-EM structure. We conclude that the majority of the KlebC_1-254_ C-terminus (~100 residues) is exposed in the complex with TolC, consistent with much of helix 2 projecting out of the tunnel. Importantly, two N-terminal trypsin sites, at positions 61 and 62, are not protected (there are no other trypsin sites in the first 60 residues of the construct). Hence, concomitant with the formation of the *Kq*TolC-KlebC_1-254_ complex, the N-terminal ~60 residues of KlebC_1-254_, which houses the bacteriocin’s TonB-box motif, likely escapes through the TolC iris to become solvent-exposed. The consequences of this binding mode are explored further in “Discussion”.

### KlebC inhibits drug efflux through TolC

**Fig. 4:**
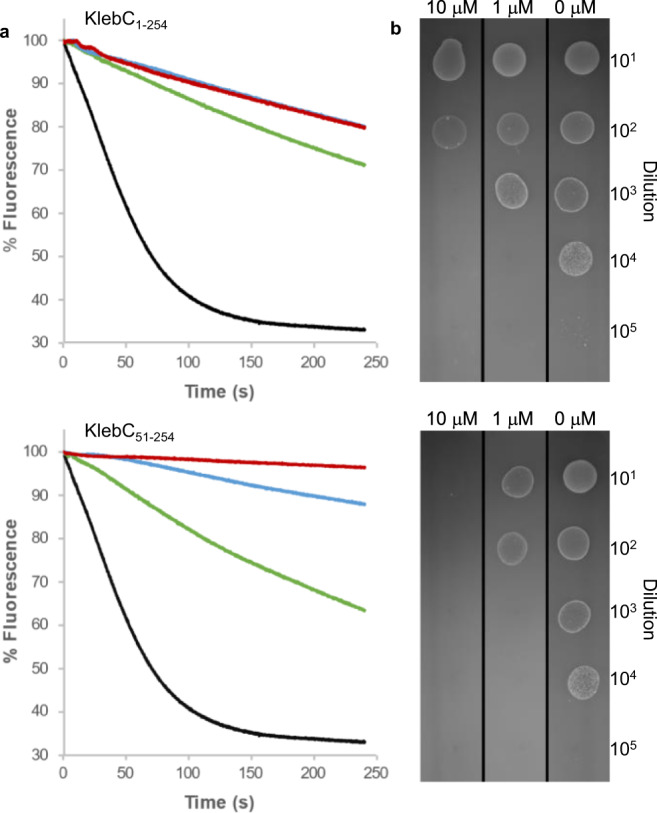
KlebC fragment-based inhibition of TolC efflux activity resulting in decreased viability of *K. quasipneumoniae*. **a** Inhibition of Nile Red Efflux through TolC in the presence of 100 µM (red), 10 µM (blue), 1 µM (green) or 0 µM (black) KlebC_1-254_ or KlebC_51-254_. Data shown are one representative experiment of two biological repeats. **b** Growth of serial dilution of *K. quasipneumoniae* prepared in the presence of 10, 1 or 0 µM KlebC_1-254_ or KlebC_51-254_ when plated onto LB-Agar in the presence of 1 mg ml^−1^ Rhodamine 6G.

We hypothesised that the high-affinity binding of KlebC_1-254_ to the *Kq*TolC lumen might impact its function as a drug efflux channel. To address this possibility, KlebC_1-254_ was added to *K. quasipneumoniae* Qmp M1-977 cells, and the ability of these cells to efflux Nile Red was monitored using fluorescence (Fig. 4). Nile Red efflux is a standard assay in studies investigating TolC-mediated export in live bacterial cells^27^. We found that 1 µM KlebC_1-254_ was sufficient to show significant inhibition of Nile Red efflux, with concentrations from 10 to 100 µM KlebC_1-254_ reaching the maximum inhibition observed. Whilst KlebC_1-254_ bound TolC with an affinity tenfold higher than KlebC_51-254_, the former contains an intact TonB-binding sequence within its unstructured N-terminus meaning that this inhibitor can be transported across the outer membrane in a TonB-dependent manner. In total, 1 µM KlebC_51-254_ inhibited Nile Red efflux, but to a lesser extent than was seen with the higher affinity inhibitor KlebC_1-254_. However, at 10 µM, inhibition afforded by KlebC_51-254_ exceeded that of KlebC_1-254_ and at 100 µM KlebC_51-254_ gave near-complete inhibition of Nile Red efflux.

TolC plays a critical role in the excretion of compounds that would otherwise be toxic to the cell^3^. *E. coli tolC* deletion strains have been shown to have increased sensitivity to numerous compounds including Rhodamine 6G, where the MIC changes from 1.28 mg ml^−1^ in wild-type strains to 2.5 µg ml^−1^ in a *tolC* deletion^28^. The impact of TolC inhibition by KlebC was assessed by preparing tenfold serial dilutions of *K. quasipneumoniae* cultures in the presence of 0, 1 or 10 µM KlebC_1-254_ or KlebC_51-254_ followed by growth on LB-Agar supplemented with 1 mg ml^−1^ Rhodamine 6G. The addition of KlebC-based inhibitors impacted the ability of *K. quasipneumoniae* to grow in the presence of Rhodamine 6G, with KlebC_51-254_ giving the most prominent inhibition, preventing growth after dilution in 10 µM inhibitor, a 4-log reduction compared to growth in its absence. We conclude that TolC-mediated drug efflux is inhibited by the N-terminal helical-hairpin domain of KlebC and that the efficacy of this inhibition is enhanced when bacteriocin translocation into cells is abolished through the removal of the TonB box.

## Discussion

In this study, we used a KlebC-E9 DNase hybrid protein to define the outer-membrane uptake pathway of the bacteriocin KlebC in *Klebsiella* spp. We demonstrated that an N-terminal 254-residue domain binds TolC with high affinity and likely uses the efflux channel both as its OM receptor and translocator since no additional outer-membrane proteins are required. TolC dependence for bacteriocin toxicity has been shown previously for *E. coli*-specific bacteriocins, ColE1, Col5 and Col10. In these instances, however, toxicity is also dependent on additional OM proteins acting as the receptor for the toxin; BtuB in the case of ColE1, Tsx for Col5 and Col10^5^. All these toxins have a TolC-binding domain at the N-terminus similar to that of KlebC (Supplementary Fig. 1e), which we show by crystallography forms a helical-hairpin spanning 150 Å and where the first ~60 residues are disordered. Disordered N-termini are a common structural feature of bacteriocins, particularly those produced by the *Enterobacteriaceae*. This region contains epitopes destined to contact energised TonB or Tol proteins in the periplasm, as in the case of KlebC which houses a TonB box. In order to deliver these epitopes to the periplasm, however, an outer-membrane translocator is required, with delivery being either active or passive. Examples of energised transfer include pyocins S2 and S5 in *P. aeruginosa*, where both toxins exploit the PMF to deliver a TonB box through their OM TBDT translocator prior to import^13,29^. Passive transfer of bacteriocin disorder epitopes includes those of colicins E2-E9 in *E. coli*, whereby contact with TolB in the periplasm is achieved by insertion of the disordered sequence at the N-termini of these toxins through the pores of the general porins OmpF or OmpC^16,30^. Our protease protection assays suggest that KlebC similarly manages to passively translocate its disordered N-terminal domain, containing the TonB box, through the iris of TolC into the periplasm.

**Fig. 5:**
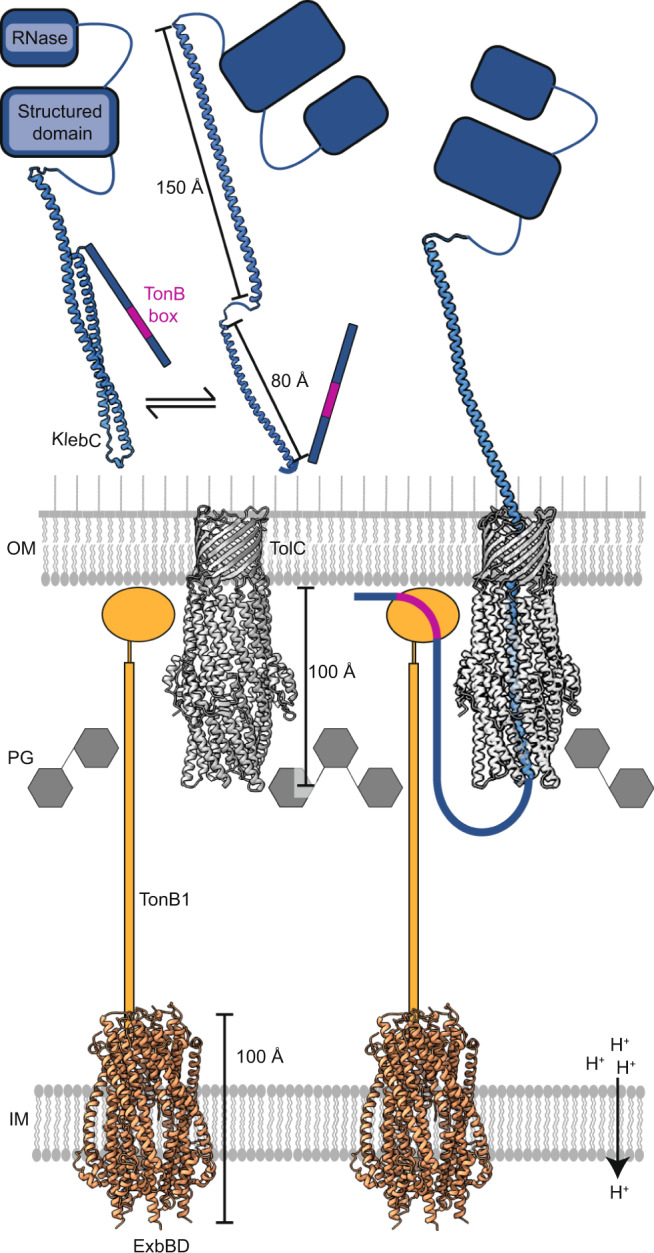
Model depicting how the KlebC toxin crosses the OM through TolC. The N-terminal domain of KlebC (blue) exists as a folded hairpin in solution, with a disordered N-terminal domain containing a TonB-box sequence (magenta). The helical hairpin is likely in equilibrium with an unfurled counterpart, which is the binding-competent form of KlebC. We speculate that electrostatic attraction brings the positively charged N-terminus of KlebC into the negatively charged TolC channel. Thereafter, KlebC snakes through the TolC channel, resulting in ultra-slow association kinetics for the complex. The disordered N-terminal region of KlebC then passes through the periplasmic entrance of TolC, which likely requires flexing of the iris helices to accommodate a disordered polypeptide. Around 70 residues at the N-terminus of KlebC escape the iris leaving the helical regions of KlebC docked within the TolC channel. The disordered nature of these residues means that a wide search radius is possible. Toxin translocation is driven by TonB, which associates with the KlebC TonB box (residues 16–20), in conjunction with the PMF-linked inner membrane stator complex ExbB-ExbD. Previous work on TonB-dependent bacteriocins has shown that this protein–protein interaction is sufficient to drive entry into the cell^13^. How the nuclease then translocates to the cytoplasm is not known but may involve the inner membrane AAA^+^ATPase/protease FtsH, as has been shown for *E. coli*-specific colicins^52, 53^.

KlebC uses a highly unusual binding mechanism to associate with its outer-membrane receptor/translocator TolC, in which its N-terminal helical-hairpin domain opens like a switchblade knife. Remarkably, this unfurling is not rate-limiting for binding to the TolC channel, as suggested by our stopped-flow experiments. We speculate that the ultra-slow on-rate for the complex instead reflects the N-terminal disordered region and helix 1 snaking through the channel following hairpin unfurling. Once bound, KlebC then likely extends its remaining domains from the cell surface (Fig. 5).

TolC constitutes the exit channel for different tripartite efflux pumps that span the two membranes of the Gram-negative cell envelope, coupling expulsion of antibiotics and toxins to either proton flow across the inner membrane or ATP hydrolysis in the cytoplasm^31^. Examples include AcrAB, EmrAB and MacAB. In each case, one protein acts as the adapter (e.g. AcrA) linking the energised protein (e.g. AcrB) in the inner membrane to the periplasmic entrance of TolC. Indeed, TolC is recruited to efflux pumps loaded with secretable substrates by their adapters (reviewed by Koronakis et al.^3^). The present work emphasises that the TolC channel can be recruited to energised inner membrane systems not involved in drug or toxin efflux. By coupling to the PMF-linked TonB/ExbBD complex, TolC acts as an OM translocation portal for bacteriocins that present a TonB-box epitope to the periplasm. TolC can also be recruited to the PMF-linked TolA/TolQR complex in the inner membrane since some group A bacteriocins, such as ColE1, present Tol protein interaction epitopes in the periplasm via TolC^5^. Finally, since the high-affinity binding of KlebC to TolC blocks drug efflux, we suggest these proteins may be the starting point for developing approaches to counter multidrug-resistant Gram-negative bacteria.

## Methods

### Molecular biology

A synthetic gene encoding heterodimeric NdeI-KlebC/ImC-XhoI codon optimised for *E. coli* expression (Eurofins genomics) could not be assembled from its three component parts due to its toxicity. Following truncation of KlebC to replace the cytotoxic domain with a NcoI site, NdeI-KlebCΔrRNase-NcoI was constructed and cloned into the NdeI/NcoI sites of pCS4^32^ to give KlebC-E9 DNase/Im9_His6_ in pET21a. N- and C-terminal truncations and site-directed mutagenesis were performed through standard PCR techniques and cloned into the NdeI/XhoI sites of pET21a or pET24a. TolC was amplified from *Klebsiella quasipneumoniae* Qmp M1-977 genomic DNA and cloned into the NdeI/XhoI sites of pET24a such that the plasmid provided a C-terminal His_6_ tag on TolC. A complete list of plasmids used in this work is given in Supplementary Table 5, and the primers used in their construction and mutagenesis are given in Supplementary Table 6.

### Protein expression and purification

TolC was expressed in T7 Express lysY/Iq cells (NEB) transformed with pNGH317 and grown in LB (Lennox) supplemented with 50 µg ml^−1^ kanamycin at 37 °C to an OD_600_ of 0.75 upon which IPTG was added to a final concentration 1 mM, followed by a further 4 h growth at 20 °C. Cells were harvested by centrifugation (5000 × *g*, 12 min, 4 °C) and cell pellets resuspended in 10 mM Tris-HCl, pH 8.0, 0.25% (w/v) lithium, 3,5-diiodosalicylic acid in the presence of 1 mM PMSF. Cells were lysed through sonication (Misonix S-4000) and insoluble cell debris removed by centrifugation (5000 × *g*, 15 min, 4 °C), before pelleting membranes from the supernatant (200,000 × *g*, 1 h, 4 °C). Outer-membrane proteins were detergent solubilised in 10 mM Tris-HCl, pH 8.0, 5 mM EDTA and 2% (w/v) β-OG, with insoluble material removed by ultracentrifugation (200,000 × *g*, 1 h, 4 °C). EDTA was removed from the supernatant using a HiPrep 26/10 desalting column (Cytiva) equilibrated in 25 mM Tris-HCl, pH 7.5, 150 mM NaCl, 1% (w/v) β-OG. Protein was purified on a 5-ml HisTrap FF column (Cytiva) equilibrated in 25 mM Tris-HCl, pH 7.5, 150 mM NaCl, 1% (w/v) β-OG, eluting bound material with a 0–500 mM imidazole gradient. Homogeneous TolC was obtained after passing the nickel column eluate down a HiLoad 16/600 Superdex 200 pg column (Cytiva) equilibrated in 25 mM Tris-HCl, pH 7.5, 150 mM NaCl, 1% (w/v) β-OG.

KlebC-E9/Im9, truncated KlebC constructs and mutants were expressed in BL21 (DE3) transformed with the appropriate plasmids and grown at 37 °C in LB (Miller) supplemented with either 50 µg ml^−1^ kanamycin or 100 µg ml^−1^ ampicillin. Cultures were grown to an OD_600_ of 0.75 upon which expression was induced by the addition of IPTG to a final concentration of 1 mM, followed by a further 4 hours growth at 28 °C before harvesting by centrifugation (5000 × *g*, 12 min, 4 °C). Cell pellets were resuspended in 20 mM Tris-HCl, pH 8.0, 500 mM NaCl, 8 mM imidazole 1 mM PMSF, lysed by sonication and clarified by centrifugation at 17,500 × *g* for 30 min at 4 °C, before passing through a 0.45-µm filter and loading onto a 5 ml HisTrap FF column (Cytiva). Bound material was eluted with an imidazole gradient to 500 mM. Fractions containing the protein of interest were pooled and buffer exchanged into 25 mM Tris-HCl, pH 7.5, 150 mM NaCl, by overnight dialysis prior to further purification on a HiLoad 26/600 Superdex 200 pg column (Cytiva) for KlebC-E9/Im9 or a HiLoad 26/600 Superdex 75 pg column (Cytiva) for truncated KlebC constructs, equilibrated in the same buffer. Proteins were quantified through the measurement of A_280_ using sequence-based extinction coefficients.

For the preparation of selenomethionine-labelled KlebC_51-254_, T7 Express Crystal competent *E. coli* (NEB) transformed with pNGH337 were used to inoculate 50 ml cultures of M9 media supplemented with 2 mM MgSO_4_, 0.1 mM CaCl_2_, 0.4% (w/v) glucose, 0.0002% (w/v) ferric ammonium acetate, 50 µg ml^−1^ l-methionine and 50 µg ml^−1^ kanamycin, which were then grown for 20 h at 37 °C. In all, 25 ml of this culture was used to inoculate 660 ml of the same media, which was grown at 37 °C to an OD_600_ of 0.7 before harvesting by centrifugation at 4000 × *g* for 15 min at 20 °C. Cell pellets were resuspended in 660 ml M9 media supplemented with 2 mM MgSO_4_, 0.1 mM CaCl_2_, 0.4% (w/v) glucose, 0.0002% (w/v) ferric ammonium acetate and 50 µg ml^−1^ kanamycin and were grown at 37 °C for 2.5 h to deplete methionine. Selenomethionine was added (50 µg ml^−1^), followed by 30 min growth at 37 °C before inducing expression with 1 mM IPTG. Cells were grown for a further 3 h at 37 °C before harvesting by centrifugation, with the remainder of the purification performed, as described above.

For the preparation of NMR samples, BL21 (DE3) cultures transformed with appropriate plasmids were grown in M9 media supplemented with 2 mM MgSO_4_, 0.1 mM CaCl_2_, 0.4% (w/v) glucose, 0.17% (w/v) yeast nitrogen base, 15 mM ^15^NH_4_Cl in the presence of 50 µg ml^−1^ kanamycin at 37 °C to an OD_600_ of 0.5, upon which IPTG was added to 1 mM and cultures were grown for a further 3 h at 37 °C. The remainder of the purification was performed as described above.

### KlebC cytotoxicity assay

Killing assays were performed using Klebsiella *quasipneumoniae* Qmp M1-977, in addition to BW25113, JW0940 (BW25113 *ompA-*), JW5503 (BW25113 *tolC-*), JW0452 (BW25113 *acrA-*), JW0451 (BW25113 *acrB-*), JW0729 (BW25113 *tolA-*) and JW5195 (BW25113 *tonB-*) from the Keio collection obtained through The Coli Genetic Stock Center, Yale. In total, 5 ml molten LB—0.7% (w/v) Agar inoculated with mid-log culture was overlaid onto 20 ml LB—1.5% (w/v) Agar in a 90-mm Petri dish and allowed to set. In all, 5 µl of 27, 9, 3, 1 and 0.3 μM KlebC were spotted onto the plate and once dry were incubated at 37 °C overnight. KlebC activity resulted in zones of clearance in the bacterial lawn.

### In vivo assembly and extraction of KlebC complexes

Two 50 ml of nutrient broth (Merck) cultures of *K. quasipneumoniae* Qmp M1-977 grown for 8 h at 37 °C were each used to inoculate 1 L of nutrient broth, which was then grown for a further 16 h at 37 °C. Cell pellets were harvested by centrifugation at 5000 × *g* at 4 °C for 12 min. Pellets were resuspended in 10 mM Tris-HCl, pH 8.0, 0.25% (w/v) lithium, 3,5-diiodosalicylic acid, 1 mM PMSF with and without 10 mg KlebC-E9/Im9_His6_. Samples were lysed through sonication followed by centrifugation at 15,000 × *g* for 15 min at 4 °C to remove cell debris. Total membranes were pelleted through centrifugation at 200,000 × *g* for 1 h at 4 °C. Inner membranes were extracted in 10 mM Tris-HCl, pH 8.0, 0.25% (w/v) lithium, 3,5-diiodosalicylic acid, 2% (v/v) Triton X-100 with outer membranes again pelleted at 200,000 × *g* for 1 h at 4 °C. Outer-membrane pellets were washed with 25 ml of 10 mM Tris-HCl, pH 8.0 to remove excess Triton X-100, followed by centrifugation at 200,000 × *g*. Outer membranes were solubilised in 10 mM Tris-HCl, pH 8.0, 5 mM EDTA, 2 % (w/v) β-OG, followed by centrifugation at 200,000 × *g*. The outer-membrane extracts were buffer exchanged into 10 mM Tris-HCl, pH 8.0, 1% (w/v) β-OG using a HiPrep 26/10 desalting column (Cytiva) before loading onto a 1-ml HisTrap HP column (Cytiva) and eluting bound material with a 0–500 mM imidazole gradient over 10 column volumes. Eluted fractions for both the control and KlebC-E9 DNase/Im9_His6_ baited sample, were analysed on a 10% SDS-PAGE gel. Protein bands unique to the sample in the presence of bait protein were subjected to in-gel digest with trypsin followed by peptide fingerprinting.

### Analytical gel-filtration chromatography

KlebC constructs and TolC were buffer exchanged into 25 mM Tris-HCl, pH 7.5, 150 mM NaCl, 1% (w/v) β-OG using a 5 ml HiTrap desalting column (Cytiva) before preparing individual components at a concentration of 5 µM and complexes containing 5 µM of each component. In all, 500 µl of each sample was injected onto a 10/300 Superdex 200 GL column (Cytiva) equilibrated in the same buffer. Analytical gel filtration was performed at a flow of 0.5 ml/min monitoring eluate at 280 nm and 220 nm. The impact of disulphide bonds on complex formation was investigated by treating KlebC-E9 DNase/Im9 with either 5 mM diamide or 5 mM DTT prior to gel filtration. The gel filtration itself was performed in 25 mM Tris-HCl, pH 7.5, 150 mM NaCl, 1% (w/v) β-OG in the absence of oxidising or reducing agents.

### Equilibrium fluorescence measurements

Equilibrium fluorescence measurements were made on a Horiba FluoroMax-4 spectrofluorimeter at 25 °C. Fluorescence spectra were measured in a 5 mm cuvette using excitation wavelengths of 280 or 295 nm collecting emissions over 300–400 nm for tryptophan emissions or 400–550 nm for AEDANS fluorescence, with excitation and emission slit widths of 2 nm.

### Pre-equilibrium fluorescence

Pre-equilibrium fluorescence measurements were performed on an SX20 stopped-flow (Applied Photophysics), using 1:1 vol:vol mixing ratio, at 25 °C. Excitation wavelengths of 280 or 295 nm were selected with a monochromator, with slit widths of 0.5 mm and emission wavelengths above 320 nm (for tryptophan fluorescence) or 445 nm (for AEDANS fluorescence) were selected with cut-off filters. Data were collected over suitable time courses (varying from 50 ms to 1000 s) under pseudo-first-order conditions. The apparent rate constant was obtained by fitting data to a single exponential equation, with *k*_on_ determined from the gradient of *k*_app_ plotted against a range of excess protein concentrations.

### Isothermal titration calorimetry

Titrations were performed on an iTC200 isothermal titration calorimeter (Malvern instruments), with proteins buffer exchanged into 25 mM Tris-HCl, pH 7.5, 1% (w/v) β-OG using a 5 ml HiTrap desalting column (Cytiva). KlebC constructs were used in the syringe at concentrations between 76 and 89 µM, whilst TolC trimer was present in the cell at concentrations between 5 and 7.6 µM. Due to the very slow kinetics of binding, data were collected in no feedback mode, with 750 s spacing between injections. Data were fitted to a single set of identical sites binding model.

### Crystallisation

Selenomethionine-labelled KlebC_51-254_ concentrated to 8.6 mg ml^−1^ using a VivaSpin20 with 10 kDa MWCO (Sartorius), was used to prepare JCSG + , PACT premier, Morpheus (Molecular Dimensions), PEG/Ion and Index (Hampton Research) commercial screens by sitting-drop vapour diffusion using 100 nl of protein mixed with 100 nl of reservoir solution, at 18 °C. Crystals obtained in 0.2 lithium sulfate, 0.1 M BisTris, pH 5.5, 25% (w/v) PEG3350 of the Index screen, cryocooled in the presence of 30% ethylene glycol gave poor-quality powdery diffraction. The remaining crystals from this condition were used to prepare a crystal seed stock using a Microseed Bead (Molecular Dimensions) following the manufacturer’s instructions. Commercial screens were again set up using sitting-drop vapour diffusion, this time with 100 nl of 8.6 mg ml^−1^ selenomethionine-labelled KlebC_51-254_, 50 nl of seed stock and 150 nl of reservoir solution. Crystals were obtained from several conditions with the best diffracting growing in Morpheus condition G1 (0.1 M Morpheus Carboxylic acids, 0.1 M Morpheus Buffer 1 pH 6.5, 30% v/v Morpheus Precipitant Mix 1). Diffraction data were collected at beamline I04, Diamond Light Source at a wavelength of 0.97932 Å to an upper-resolution limit of 1.9 Å. Data reduction was carried out using DIALS^33^ and a substructure of three Se atoms was determined using SHELXD^34^ and refined using PHASER^35^. An initial model was built automatically using BUCANNEER^36,37^ and was completed and refined using iterative cycles of COOT^38^ and BUSTER^39^. Data collection and refinement statistics are presented in Supplementary Table 1. The atomic coordinates and structure factors for KlebC_51-254_ have been deposited in the Protein Data Bank (PDB ID code 7NNA).

### Liquid growth-killing assay

An overnight culture of *K. quasipneumoniae* Qmp M1-977 was used to inoculate 100 ml LB (Miller) which was grown at 37 °C to an OD_600_ of ~0.2 upon which 10 ml aliquots were removed and treated with 100 nM KlebC-E9/Im9, 100 nM A107C, Y177C KlebC-E9/Im9 oxidised with 5 mM diamide, 100 nM A107C, Y177C KlebC-E9/Im9 reduced with 5 mM DTT, 100 nM L86C, L198C KlebC-E9/Im9 oxidised with 5 mM diamide, 100 nM L86C, L198C KlebC-E9/Im9 reduced with 5 mM DTT or no klebicin. Cultures were grown for a further 2 h monitoring OD_600_ every 30 min.

### Preparation of KlebC–TolC complexes in Amphipol A8-35

KlebC_1-254_ was added to TolC in a twofold molar excess and the complex was buffer exchanged into 20 mM potassium phosphate, pH 7.5, 100 mM NaCl, 1% (w/v) β-OG using a HiPrep 26/10 desalting column (Cytiva). Formaldehyde was added to the complex at a final concentration of 1% (w/v) and incubated at room temperature for 30 min before quenching with 100 mM Tris-HCl. Cross-linked KlebC_1-254_·TolC complex was purified on a HiLoad 16/600 Superdex 200 pg column (Cytiva) equilibrated in 20 mM potassium phosphate, pH 7.5, 100 mM NaCl, 1% (w/v) β-OG. In total, 10 mg of amphipol A8-35 (Anatrace) was added for each mg of protein and incubated at room temperature for 2 h. In all, 10 mg of BioBeads SM2 (Bio-Rad) were added to the sample per mg of detergent, incubated for 2 h before centrifuging at 20,000 × *g* for 5 min at room temperature. Formaldehyde cross-linked KlebC_1-254_·TolC complex in amphipol A8-35 was purified on a HiLoad 16/600 Superdex 200 pg column (Cytiva) equilibrated in 20 mM potassium phosphate, pH 7.9, 100 mM NaCl. Eluate was concentrated to 1.7 mg ml^−1^ using a VivaSpin Turbo 4, 50 kDa (Sartorius) concentrator.

### Single-particle Cryo-EM data collection

*Kq*TolC-KlebC_51-245_ complex was applied to Quantifoil 300 mesh Cu, 1.2/1.3, grids at concentrations of 0.5 mg ml^−1^ and 0.75 mg ml^−1^ for native and formaldehyde cross-linked samples, respectively. Samples (3 μl) were vitrified using a vitrobot Mark IV (Thermo Fisher) with a blot time of 3 s, blot force of −2, and wait time of 30 s. Cryo-EM data were acquired on a 300-kV Titan Krios microscope, equipped with a K2 detector (Gatan), recording in counting mode. A pixel size of 0.822 Å was used to collect videos of 30 frames with a dose rate of 4.8 and 3.7 electrons/Å^2^/s, from an 8-s exposure, resulting in a total dose of 38 and 29 electrons/Å^2^ for native and formaldehyde cross-linked samples, respectively. For further collection parameters, see Supplementary Table 2.

### Cryo-EM data processing and model building

Initially, motion correction (MotionCorr 2) and CTF estimation (ctffind 4) were carried out using the SIMPLE pipeline^40^. A subset of micrographs (20) was manually picked to generate templates for automatic picking within SIMPLE. Following picking the particle set was cleaned up using 2D classification, after which Relion-3.0 and Relion-3.1^41–43^, were used for further processing. Particles (337,277 for the native and 626, 767 for the cross-linked sample) were sorted using 3D classification with three classes. For the uncrosslinked TolC sample, one rubbish class was generated with two other classes appearing similar, thus particles from these classes were combined and processed further with 3D refinement. A final map of TolC without KlebC present extending to 3.4 Å, was generated after detergent subtraction, CTF refinement and map sharpening. Processing is summarised in Supplementary Fig. 5.

For the cross-linked TolC-KlebC_1-254_ sample, a single 3D class was selected for further processing using 3D refinement. Following refinement, the resolution of the map extended to 5.5 Å therefore, a second 3D classification step was implemented to sort heterogeneity. Of the three resulting classes, a single class displayed clear additional density present within the TolC β-barrel, which was attributed to KlebC. Refinement of this class gave a 3.4 Å map, with three short KlebC helices located within the β-barrel of TolC. The pseudo C3 symmetry apparent in this map led to the re-refinement of the data with C3 symmetry. Subtraction of amphipol shell, CTF refinement and post-processing were subsequently carried out to generate a 2.8 Å TolC-KlebC map. Since biophysical data suggested that only a single KlebC molecule bound the TolC trimer, the particles were expanded using the C3 symmetry and subtracted to give a single TolC monomer. 3D classification of monomeric particles into eight classes without alignment gave three classes that had density consistent with both TolC and KlebC. In these classes, the KlebC density extended past the β-barrel domain of TolC into the lumen. Reverse subtraction and refinement of the classes a final map from one subclass, with KlebC density that spanned the length of the TolC lumen. After iterative rounds of model building and LocScale^44^ sharpening, the KlebC density within the final map was more clearly resolved.

An atomic model was built for *Kq*TolC after docking of *E. coli* TolC (1EK9) into the native *Kq*TolC map in chimera^45^ and modification of the sequence within coot^46^ to match that of *Kq*TolC. Iterative rounds of real-space refinement and manual model building were implemented in phenix^47–49^ and coot, respectively, to yield final models with statistics reported in Supplementary Table 2 and map to model fits Supplementary Fig. 6. To build a model of the *Kq*TolC-KlebC complex, refined *Kq*TolC was docked into the final 3.1 Å map in chimera. After a round of rigid-body fitting and manual model building in Phenix and Coot, respectively, KlebC was built in de novo, after which the fit was improved through iterative rounds of real-space refinement, manual model building and map sharpening in Phenix, Coot and LocScale, respectively. Due to the weak density corresponding with the KlebC portion of the map, helix 2 was built within coot by modelling two short alanine helices with opposing register. From this dual modelling, the helical register was apparent favouring one conformation and side chains were iteratively mutated, to residues within the KlebC sequence that best fit the unmodelled KlebC density (Supplementary Fig. 6k). Helix 1 was subsequently placed into the remaining density, once again modelled as two polyalanine helices with opposing register. The helix that best fit the density was consistent with that independently assigned to helix 2. The sequence connecting helix 1 and 2 was built *de novo*. The final model of KlebC was subsequently cross-validated by alignment to crystal structure model. Final models of *Kq*TolC and *Kq*TolC-KlebC_1-254_ complex uploaded to PDB with access codes 7NG9 and 7NG8, respectively.

### Solution NMR of KlebC_1-254_ and KlebC_51-254_

All NMR experiments were collected at 20 °C using ^15^N-labelled 250 μM KlebC_1-254_ and 250 μM KlebC_51-254_ in 95% H_2_O/5% D_2_O 25 mM Tris-HCl, pH 7.5, 150 mM NaCl. Data were collected using a 750 MHz spectrometer equipped with an Avance III HD console and a 5 mm TCI CryoProbe. Resonance assignments for KlebC_1-254_ and KlebC_51-254_ were obtained using 2D ^1^H-^15^N BEST-TROSY experiments and 3D ^15^N-edited NOESY-HSQC and ^15^N-edited TOCSY-HSQC. Backbone dynamics were monitored using the {^1^H}-^15^N heteronuclear NOE experiment.

### Trypsin digestion of the KlebC_1-254_-*Kq*TolC Complex

In total, 20 µg of sequencing grade trypsin (Promega) was added to 1 ml of 8 µM KlebC_1-254_-*Kq*TolC complex in 50 mM ammonium acetate pH 7.5, 1% (w/v) β-OG and was incubated for 2 h at 37 °C, before the addition of 2 mM PMSF and purification on a 10/300 Superdex 200 GL column (Cytiva). The elution peak of *Kq*TolC in complex with KlebC fragments was analysed on a 10–20% Tricine SDS-PAGE gel (Invitrogen). The five bands of molecular weights lower than TolC were excised from the gel, digested with trypsin and identified through peptide fingerprinting.

Gel bands were reduced with Tris(2-carboxyehtyl) phosphine (TCEP) and alkylated with chloroacetamide. Following treatment, bands were washed with acetonitrile (100%) and protein digested overnight with sequencing grade trypsin (100 ng) at 37 °C. Peptides were loaded onto reverse-phase C18 column and injected onto a Q-Exactive Orbitrap mass spectrometer (Thermofisher Scientific). MaxQuant^50^ was used to search for peptides against an *E. coli* database, with *Kq*TolC and KlebC sequences annotated within the FASTA. Data were sorted based on the intensity of peptides, with noise being defined as an order of magnitude cut-off from the highest intensity peptide identified within the sample. Within this intensity range only *Kq*TolC and KlebC peptides were sequenced.

### Native-state mass spectrometry of trypsin-digested KlebC_1-254_-*Kq*TolC complex

Native-state mass spectrometry was carried out on a Thermo Q-Exactive UHMR Quadrupole-Orbitrap platform. The protein solutions (1–3 µl, 24 µM) were loaded into gold-coated borosilicate electrospray ionisation capillary pulled in-house^51^. An electrospray was generated by elevating the capillary voltage 1.0–1.2 kV above the instrument orifice (100–150 °C). Typical instrument parameters used: resolution of 12,500 (at *m/z* 200); S-lens RF 150%; 200 ms fixed injection time; 200–250 V HCD energy at a pressure setting of 8.0. Datasets were plotted in OriginPro v. 9.5.1.195 (Northampton, MA). No background subtraction or smoothing was employed.

### Nile Red efflux inhibition assay

The inhibition of Nile Red efflux through *Kq*TolC by the addition of KlebC fragments was investigated using a method adapted from Bohnert et al.^27^. In total, 10 ml of *K. quasipneumoniae* Qmp M1-977 overnight culture grown in LB (Miller) were pelleted at 3000 × *g* for 10 min at room temperature. Cell pellets were resuspended in 20 mM potassium phosphate, pH 7.0, 1 mM MgCl_2_, followed by repeat centrifugation. Pellets were resuspended in 20 mM potassium phosphate, pH 7.0, 1 mM MgCl_2_ adjusting the volume to give an OD_600_ of 1. Aliquots (2 ml) were transferred into 15-ml Pyrex centrifuge tubes, and after 15 min carbonyl cyanide 3-chlorophenylhydrazone was added to a final concentration of 100 µM. After a 15 min incubation at room temperature, inhibitor was added to a concentration of 0, 1, 10 or 100 µM, after a further 15 min Nile Red was added to a final concentration of 5 µM. Samples were incubated for 3 h at 37 °C with shaking, followed by 1 hour at room temperature without shaking. Samples were centrifuged at 3000 × *g* for 5 min at room temperature with pellets resuspended in 2 ml of 20 mM potassium phosphate, pH 7.0, 1 mM MgCl_2_ supplemented with inhibitor as appropriate.

Nile Red fluorescence was monitored on a Horiba FluoroMax-4 spectrofluorimeter using an excitation wavelength of 552 nm, an emission wavelength of 636 nm, slit widths of 10 nm at 25 °C. In total, 300 µl sample was diluted into 2.7 ml 20 mM potassium phosphate, pH 7.0, 1 mM MgCl_2_ and fluorescence emissions monitored for 60 s. Glucose was added to a final concentration of 50 mM and fluorescence emissions recorded for a further 240 s. Fluorescence intensities were normalised such that the intensity immediately after glucose addition was taken a 100%.

### Rhodamine 6G toxicity assay

To investigate the impact TolC inhibition has on the ability of *K. quasipneumoniae* Qmp M1-977 growth in the presence of Rhodamine 6G, tenfold serial dilutions of overnight culture were prepared in 0, 1 or 10 µM inhibitor. In all, 5 µl of each dilution was spotted onto an LB-Agar plate containing 1 mg ml^−1^ Rhodamine 6G and plates were incubated overnight at 37 °C. Plates were imaged on a Syngene G:Box gel documentation system illuminating with Red LEDS in combination with UV-6 cut-off filter.

### Reporting summary

Further information on research design is available in the Nature Research Reporting Summary linked to this article.

## Supplementary information

Supplementary Information

Reporting Summary

## Data Availability

The data supporting the findings of the study are available in the article and its Supplementary Information. The atomic coordinates and structure factors of KlebC_51-254_, *Kq*TolC and the *Kq*TolC·KlebC_1-254_ complex have been submitted to the protein structure data bank (PDB ID codes 7NNA, 7NG9 and 7NG8, respectively). Electron microscopy density maps have been submitted to the EMDB with accession codes EMD-12310 and EMD-12309 for *Kq*TolC and *Kq*TolC·KlebC_1-254_, respectively. Source data are provided with this paper.

## Source data

Source Data
