# Supplementary material for: Toxin import through the antibiotic efflux channel TolC
Source: Nat Commun. 2021 Jul 30;12:4625. doi: 10.1038/s41467-021-24930-y (PMC8324772; doi:10.1038/s41467-021-24930-y)
Supplement: Supplementary file 1 — Supplementary Information [file 41467_2021_24930_MOESM1_ESM.pdf]

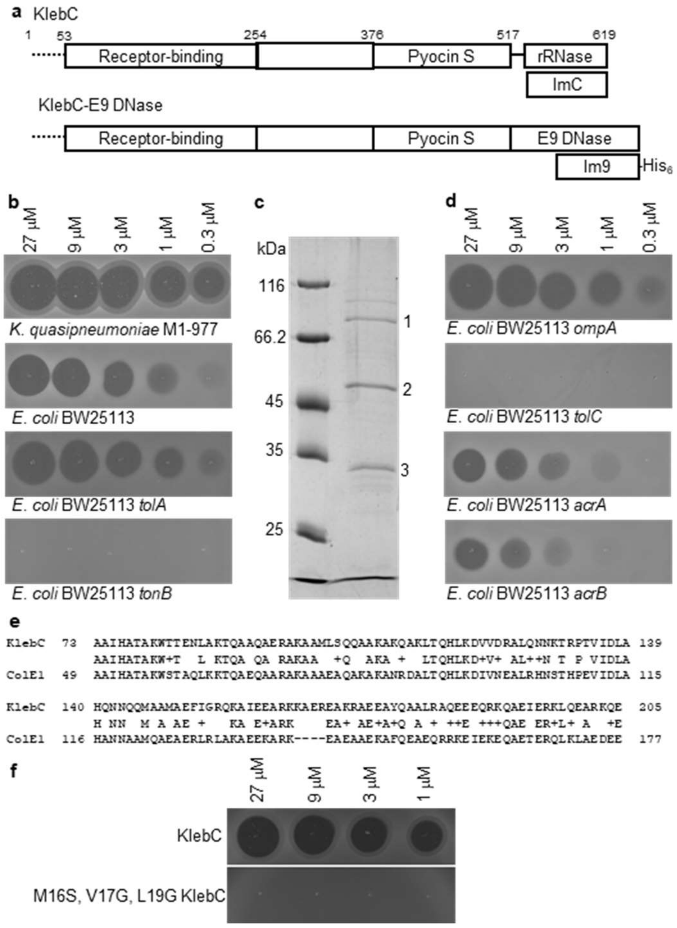

Supplementary Fig. 1. Cytotoxic activity of KlebC. **a,** Domain organisation of KlebC and the KlebC-E9 DNase hybrid. The initial 53 residues of KlebC are predicted to be intrinsically unstructured (*dashed line*), followed by a 200 residue α-helical domain. Residues 376 to 517 form a so-called Pyocin S domain, which is implicated in inner membrane translocation, followed by a ribosomal RNase domain at the C-terminus. In KlebC-E9 DNase the rRNase is replaced by the HNH endonuclease domain of colicin E9[53]. **b,** Cytotoxicity of 27, 9, 3, 1 and 0.3 µM KlebC-E9 DNase spotted onto soft-agar lawns inoculated with *K. quasipneumoniae* Qmp M1-977, BW25113, BW25113 *tolA*, or BW25113 *tonB*. **c,** affinity purified KlebC-E9 DNase/Im9 complexes assembled on and extracted from the surface of *K. quasipneumoniae*. Representative SDS-PAGE shown from n=2 biologically independent experiments. Bands 1, 2 and 3 were identified as KlebC-E9 DNase, TolC and OmpA respectively. **d,** Cytotoxicity of 27, 9, 3, 1 and 0.3 µM KlebC-E9 DNase spotted onto soft-agar lawns inoculated with BW25113 *ompA*,

BW25113 *tolC*, BW25113 *acrA*, or BW25113 *acrB*. **e,** sequence alignment of the KlebC TolC-binding domain and the ColE1 TolC-binding domain, showing 55 % sequence identity. **f,** Mutation of the putative KlebC TonB box (M16S, V17G, L19G) abolished cytotoxicity (see also Fig S3). Panel shows wild-type and mutant KlebC-E9 DNase (27, 9, 3, and 1 µM) spotted onto soft-agar lawns inoculated with *K. quasipneumoniae* Qmp M1-977.

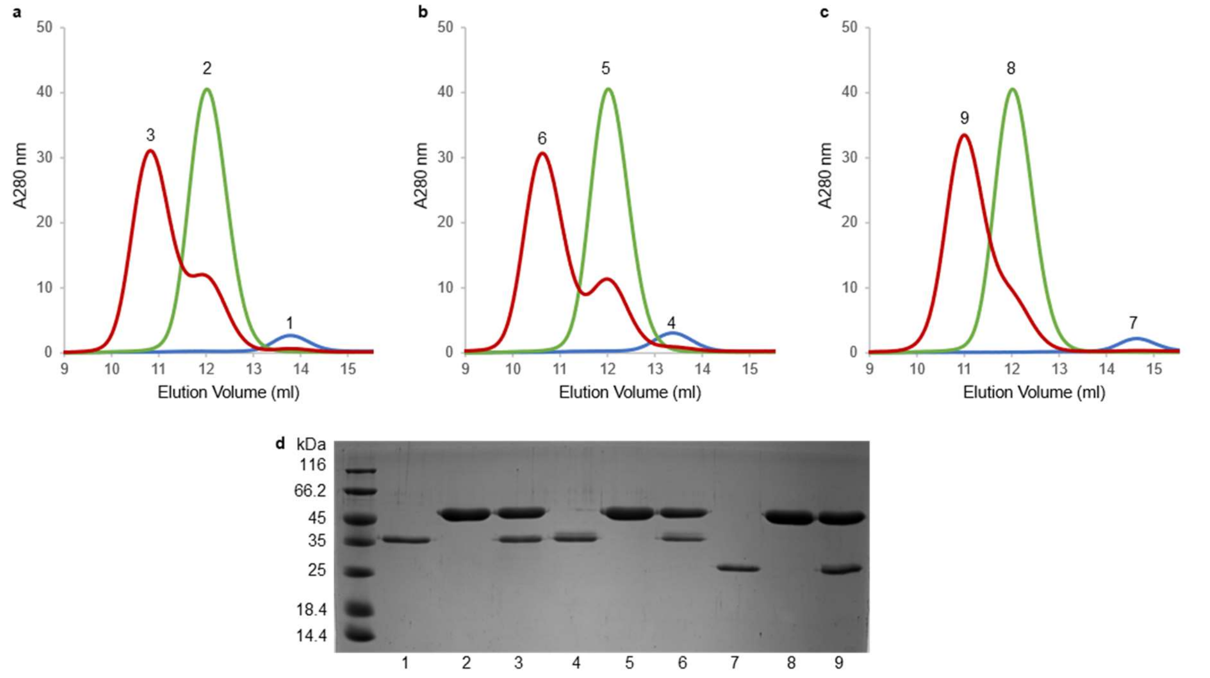

Supplementary Fig. 2. Analytical gel filtration of KlebC-*Kq*TolC complexes. 500 µl samples were analysed on a 10/300 S200 column equilibrated in 25 mM Tris-HCl, pH 7.5, 150 mM NaCl, 1 % (w/v) β-OG. **a,** 5 µM KlebC$_{1\text{-}271}$ (*blue*), 5 µM *Kq*TolC (*green*) and 5 µM KlebC$_{1\text{-}271}$ + 5 µM *Kq*TolC (*red*) A$_{280}$ elution profiles. **b,** 5 µM KlebC$_{1\text{-}254}$ (*blue*), 5 µM *Kq*TolC (*green*) and 5 µM KlebC$_{1\text{-}254}$ + 5 µM *Kq*TolC (*red*) A$_{280}$ elution profiles. **c,** 5 µM KlebC$_{51\text{-}254}$ (*blue*), 5 µM *Kq*TolC (*green*) and 5 µM KlebC$_{51\text{-}254}$ + 5 µM *Kq*TolC (*red*) A$_{280}$ elution profiles. **d,** 13.5 % SDS-PAGE analysis of elution peaks. Representative gel shown from n=2 independent experiments.

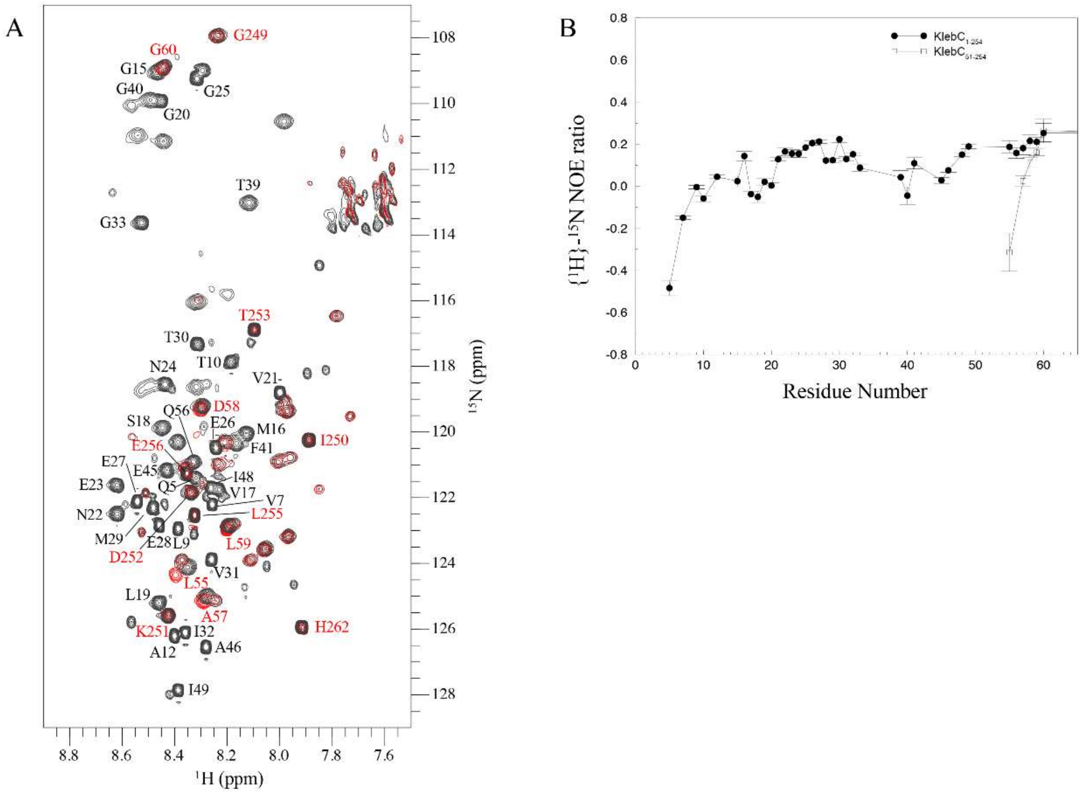

Supplementary Fig. 3. Heteronuclear NMR analysis of KlebC$_{1-254}$ and KlebC$_{51-254}$. a, Comparison of the NMR spectra of KlebC$_{1-254}$ and KlebC$_{51-254}$. Overlay of the central region of the 750 MHz Best-TROSY spectra of KlebC $_{1-254}$ (*black*) and KlebC $_{51-254}$ (*red*); peaks arising from disordered residues of proteins are typically observed in this region of the spectrum. Peaks observed in the spectra of both proteins are labeled in red; these correspond to residues 55-60 at the N-terminus of KlebC$_{51-254}$ and C-terminal residues as well as part of the His-tag. The spectrum of KlebC$_{1-254}$ contains additional strong peaks in this region, many of which have been assigned to residues 5-49 at the N-terminus of the construct. Peaks in the spectra of both constructs have been assigned using 3D ^{15}N-edited TOCSY-HSQC and NOESY-HSQC experiments collected at 750 MHz. **b,** The {^{1}H}-^{15}N heteronuclear NOE ratio for the N-terminal residues of KlebC$_{1-254}$ and KlebC$_{51-254}$ collected at 750 MHz provides information about backbone dynamics on a timescale (ps) faster than the overall tumbling of the molecule (ns). The {^{1}H}-^{15}N NOE was calculated as the ratio of the peak intensities in spectra recorded with and without ^{1}H saturation. Uncertainties in the {^{1}H}-^{15}N NOE ratios were estimated from 500 Monte Carlo simulations using the baseline noise as a measure of the error in the peak heights. A {^{1}H}–^{15}N NOE ratio of ~0.7-

0.8 is characteristic of a rigid, structured protein backbone while ratios of less than 0.6 indicate a more flexible backbone. $\{^{1}H\}$–^{15}N NOE ratios of less than ~0.3, characteristic of a highly dynamic backbone, were found for residues 55-60 of $KlebC_{51\text{-}254}$; this is consistent with the absence of electron density for these residues in the X-ray diffraction data. $\{^{1}H\}$–^{15}N NOE ratios of less than ~0.3 were also found for residues 5-60 of $KlebC_{1\text{-}254}$. The lack of chemical shift dispersion for the $^{1}H^{N}$ peaks and the low heteronuclear NOE ratios for residues 5-60 are consistent with a disordered N-terminus in $KlebC_{1\text{-}254}$. TonB box residues ($M^{16}VSLG^{20}$) are located within the disordered region of KlebC.

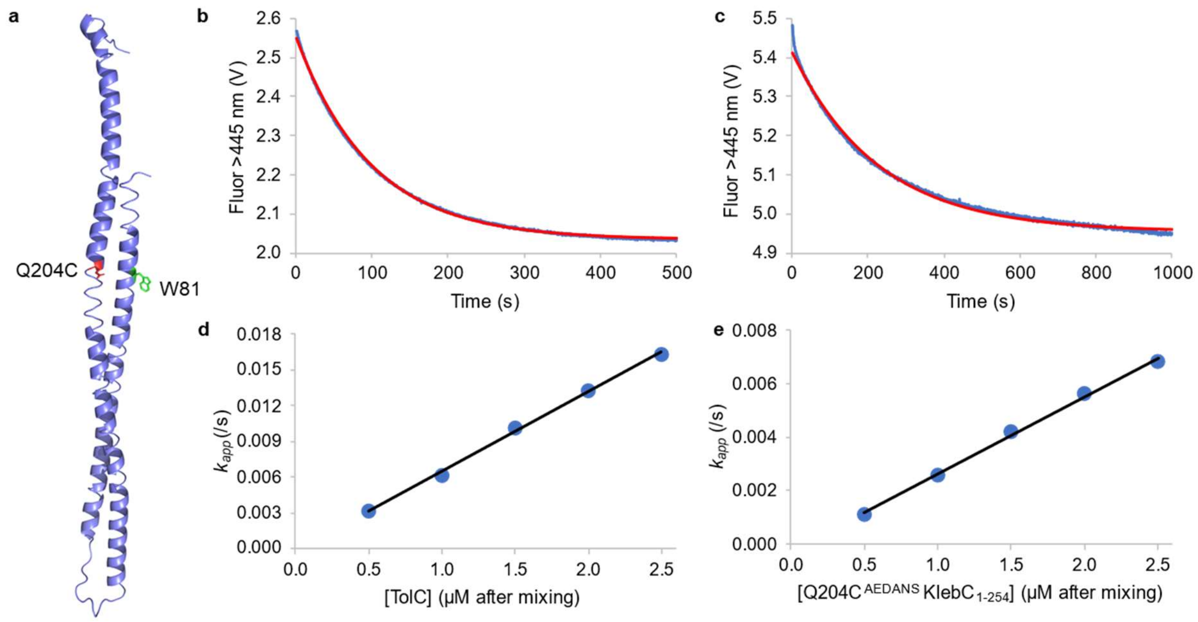

Supplementary Fig. 4. Pre-equilibrium Tryptophan-to-AEDANS FRET changes upon complex formation between *Kq*TolC and Q204C^{AEDANS} KlebC$_{1\text{-}254}$. a, Cartoon representation of KlebC$_{51\text{-}254}$ showing the sidechain of Trp81 in green and Gln204 mutated to Cys for labelling with IAEDANS shown in red. **b,** Loss of Trp-to-AEDANS FRET signal upon 0.5 µM *Kq*TolC binding 0.125 µM Q204C^{AEDANS} KlebC$_{1\text{-}254}$ with λ_{Ex}, 280 nm, $\lambda_{Em} \geq 445$ nm, slit widths of 0.5 mm, at 25 ˚C over 1000 seconds. Data were fitted to a single exponential equation to measure k_{app}. **c,** Loss of Trp-to-AEDANS FRET signal upon 0.125 µM *Kq*TolC binding 0.5 µM Q204C^{AEDANS} KlebC$_{1\text{-}254}$ with $\lambda_{Ex,}$ 280 nm, $\lambda_{Em} \geq 445$ nm, slit widths of 0.5 mm, at 25 ˚C over 500 seconds. Data were fitted to a single exponential equation to measure k_{app}. **d,** Concentration dependence of k_{app} with varied concentrations of *Kq*TolC in excess. From the gradient of the plot the association rate constant is 6.7 ± 0.2 x10^{3} M^{-1}.cm^{-1}. **e,** Concentration dependence of k_{app} with varied concentrations of Q204C^{AEDANS} KlebC$_{1\text{-}254}$ in excess. From the gradient of the plot the association rate constant is 2.9 ± 0.1 x10^{3} M^{-1}.s^{-1}. Typical traces are shown and values of association rate constants are from triplicate experiments.

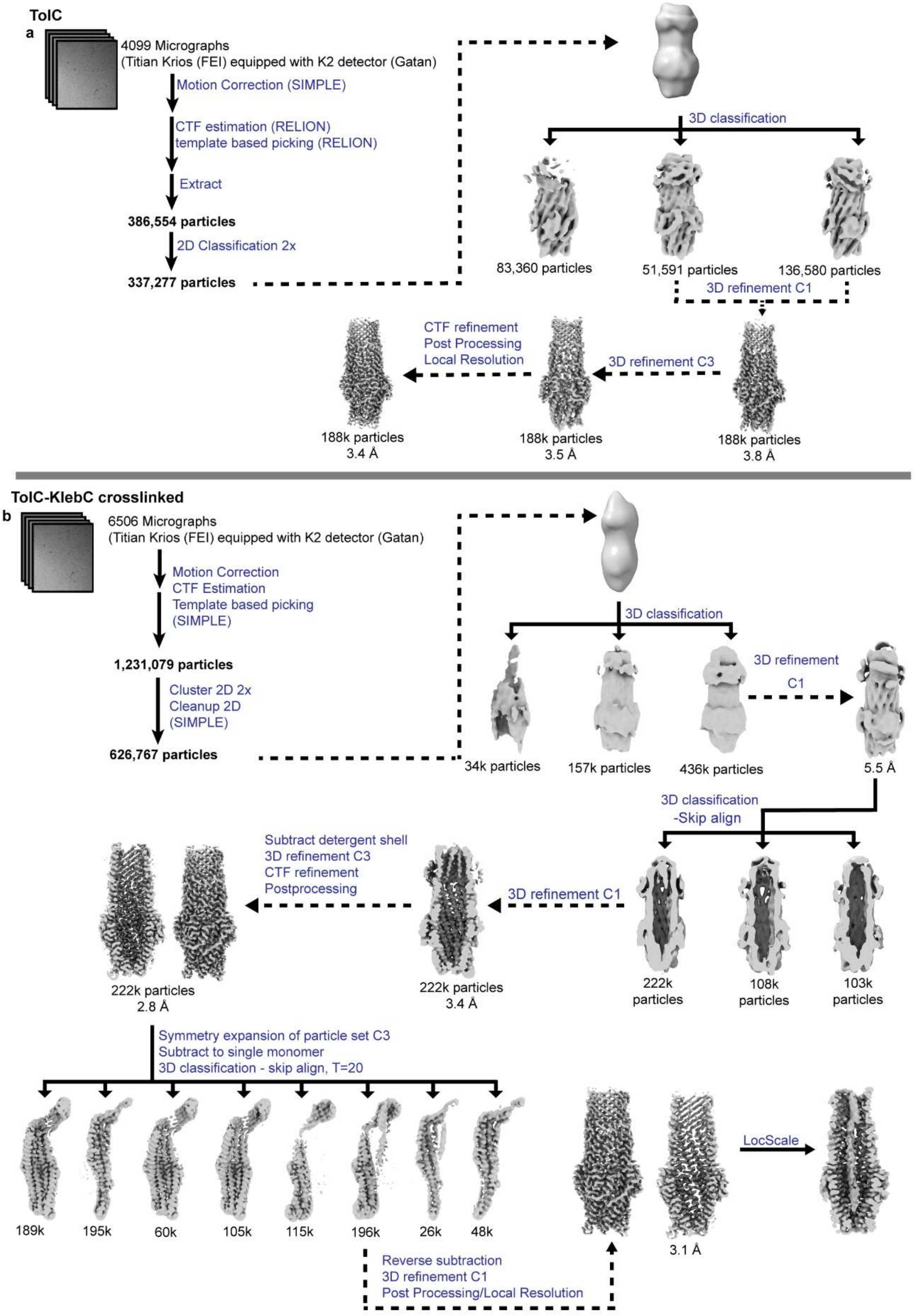

Supplementary Fig. 5. Single particle cryo-EM workflow to generate trimeric *Kq*TolC map and *Kq*TolC-KlebC$_{1\text{-}254}$ complex map. The flow chart summaries the processing steps and programs used for generation of native *Kq*TolC and *Kq*TolC-KlebC$_{1\text{-}254}$ complex maps.

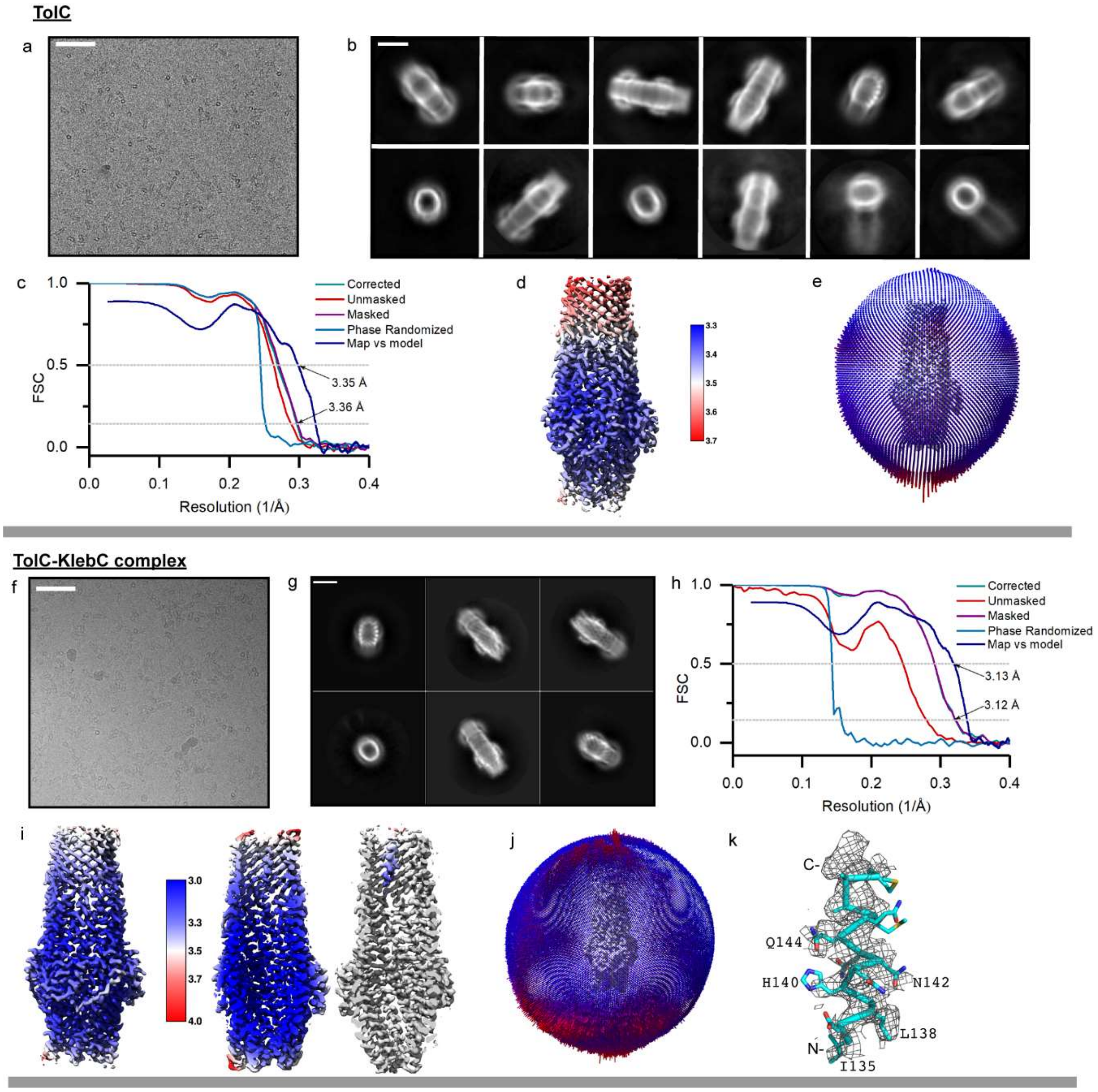

Supplementary Fig. 6. Cryo-EM map and model generation. **a**, Representative image (1/4099) of *Kq*TolC (0.5 mg.ml^{-1}) in amphipol (scale bar 500 Å). **b**, Representative reference-free 2D class averages (12/50) that were used in reconstruction of the native *Kq*TolC map (scale bar 50 Å). **c**, FSC plots for corrected (*teal*), unmasked (*red*), masked (*purple*), and phase-randomised (*blue*) maps generated during Relion post processing. The model-to-map FSC fit from phenix refinement (*navy*) is also shown. **d**, Final filtered *Kq*TolC map coloured by local resolution (Relion). **e**, Angular distribution plot from Relion refinement displayed with *Kq*TolC map (*grey*) shows a range of views with a slight preference

for periplasmic-upwards views. **f**, Representative image (1/6506) of *Kq*TolC-KlebC$_{1-254}$ formaldehyde crosslinked complex (0.75 mg.ml^{-1}) (scale bar 500 Å). **g**, Representative reference-free 2D class (6/50) averages contributing to generation of the final map (scale bar 50 Å). **h**, FSC plots coloured as in panel C, generated during post processing and phenix refinement of *Kq*TolC-KlebC$_{1-254}$ complex. **i**, from left to right *Kq*TolC-KlebC$_{1-254}$ complex map, a cut-through of the map coloured by local resolution (Relion) and a cut-through of the map with *Kq*TolC in grey and KlebC$_{1-254}$ coloured by local resolution (Relion). **j,** Angular distribution plot from Relion refinement displayed with *Kq*TolC-KlebC$_{1-254}$ complex map (*grey*) some weak preferential orientation is observed. **k**, KlebC helix 2 map density shown as a grey mesh with built model represented as sticks coloured by element.

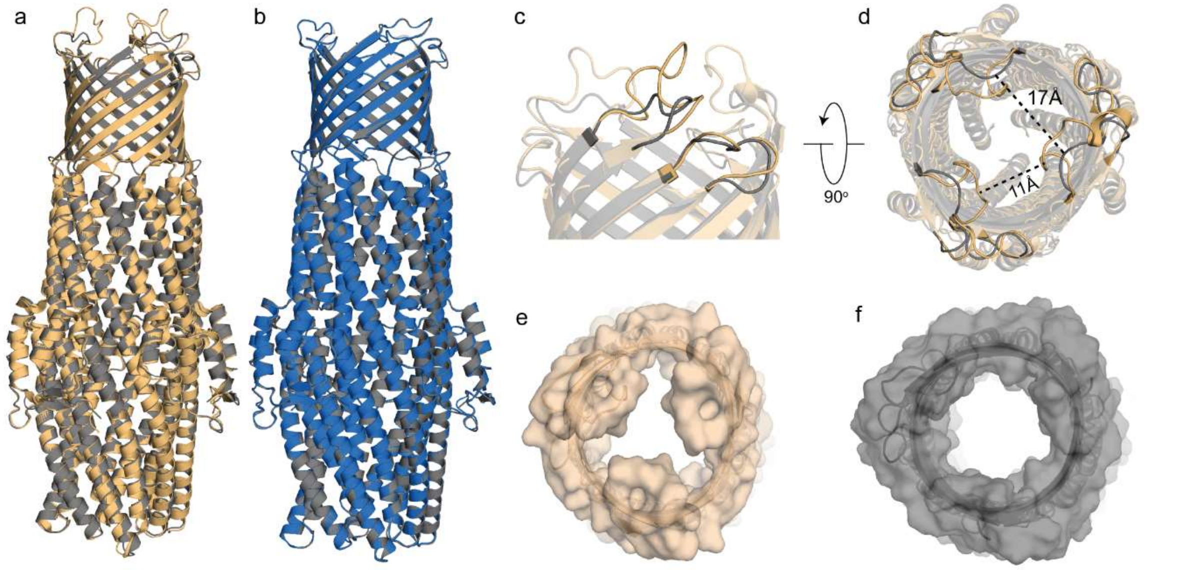

Supplementary Fig. 7. ***K. quasipneumoniae*** **TolC channel is more accessible than** ***E. coli*** **TolC due to truncation of extracellular loops.** **a**, Overlay of *K. quasipneumoniae* TolC (present work, *grey*) with *E. coli* TolC (*gold*, PDB 1EK9) shows no global differences in structure. **b**, Overlay of wild-type *Kq*TolC (*grey*) with *Kq*TolC-KlebC$_{1\text{-}254}$ (*blue*) shows no structural changes are observed upon binding of KlebC. **c-d**, Overlay of *K. quasipneumoniae* TolC (*grey*) with *E. coli* TolC (*gold*), zoomed in on the β-barrel, to highlight the extended extracellular loops in the *E. coli* structure. View in panel c is rotated 90° in panel d to look down the barrel, highlighting the increased diameter of the channel opening from 11 to 17 Å in *Kq*TolC. **e** and **f**, Extracellular facing surface representations of the β-barrels of *E. coli* TolC (gold) and Kq TolC (grey), respectively.

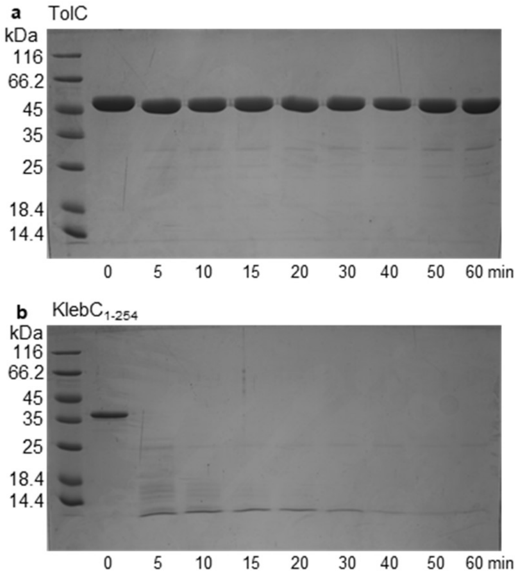

Supplementary Fig. 8. Susceptibility of *Kq*TolC and KlebC$_{1\text{-}254}$ to trypsin proteolysis. a, 12 % SDS-PAGE analysis of *Kq*TolC digested with trypsin over 1 hour. **b,** 12 % SDS-PAGE analysis of KlebC$_{1\text{-}254}$ digested with trypsin over 1 hour. Representative gels are shown from n=2 independent experiments.

	$KlebC_{51-254}$
**Data collection**	
Space group	P 3_2 2 1
Cell dimensions	
a, b, c (Å)	47.91, 47.91, 200.49
α, β ,γ (°)	90, 90, 120
Resolution (Å)	40.63 – 1.90 (1.94-1.90)*
R_{merge}	0.050 (0.433)
I/σI	14.3 (3.1)
CC(1/2)	0.997 (0.913)
Completeness (%)	99.9 (99.9)
Multiplicity	4.8 (4.9)
**Refinement**	
Resolution (Å)	1.90
No. reflections	22045
R_{work} / R_{free}	0.1884 / 0.2017
No. atoms	
Protein	1685
Water	360
Average B-factor	38.50
R.m.s deviations	
Bond lengths (Å)	0.015
Bond angles (°)	1.06

Supplementary Table 1. Data collection and refinement statistics for $KlebC_{51-254}$ crystal structure. *Denotes highest resolution shell. Data above 1.9 Å were highly anisotropic. Although diffraction was observed to a higher resolution along one axis, inclusion of these data in refinement resulted in a worse model as determined by an elevated difference between R and R_{free}.

	***Kq*TolC**<br>EMD-12310<br>PDB-7NG9	***Kq*TolC-KlebC complex**<br>EMD-12309<br>PDB-7NG8
**Data collection**		
Microscope	Titan Krios (Cosmic)	Titan Krios (Cosmic)
Voltage (kV)	300	300
Detector	Gatan K2	Gatan K2
Recording mode	Counting	Counting
Magnification	190,000	190,000
Movie/micrograph pixel size (Å)	0.822	0.822
Dose rate (e-/Å^2/sec)	4.8	3.7
Number of frames per movie	30	30
Movie exposure time (s)	8	8
Total dose (e-/Å^2)	38.4	29.6
Defocus range (um)	-0.5 to -2	-0.8 to -2.0
**EM data processing**		
Number of movies/micrographs	4099	6506
Box size (px)	216	300
Particle number (total)	386,554	1,231,079
Particle number (post 2D)	337,277	626,767
Particle number (post 3D)	188,171	435,603
Particle number (used in final map)	188,171	196,217
Symmetry	C3	C1
Map resolution (FSC 0.143)	3.3	3.12
Local resolution range (FSC 0.5)	3.3-3.7	3.0-4.0
Map sharpening B-factor (Å^2)	-185	-123
**Model Building and Validation**		
Initial model used	1EK9	7NG9
Model composition		
Non-hydrogen protein atoms	19377	20237
Protein residues	1263	1341
Nucleotides (RNA)	0	0
RMSD from ideal		
Bond length (Å)	0.006	0.005
Bond angles (°)	0.473	0.561
Validation		
Molprobity score	1.43	1.35
Clashscore	4.13	6.42
Rotamers outliers (%)	0.85	0.09
FSC (0.5) model-vs-map	3.35	3.3
CC model-vs-map (masked)	0.84	0.87
Ramachandran plot		
Favoured (%)	96.42	98.42
Allowed (%)	3.58	1.50
Outliers (%)	0	0

Supplementary Table 2. Data collection and refinement statistics for single-particle cryo-EM models of *Kq*TolC and *Kq*TolC-KlebC$_{1\text{-}254}$ complex.

Peak	Complex	Theoretical (Da)	Experimental (Da)
1	TolC + $KlebC_{81-168}$	157,571	157,606
2	TolC + $KlebC_{63-155}$	157,870	157,862
3	TolC + $KlebC_{81-172}$	158,056	158,060
4	TolC + $KlebC_{63-157}$	158,126	158,165
5	TolC + $KlebC_{67-163}$	158,338	158,317
6	TolC + $KlebC_{67-164}$	158,466	158,469
7	TolC + $KlebC_{67-165}$	158,594	158,610
8	TolC + $KlebC_{63-163}$	158,796	158,762
9	TolC + $KlebC_{63-164}$	158,924	158,904

Supplementary Table 3. Identification of heterogeneous species within the trypsin digested $KlebC_{1-254}$-*Kq*TolC complex native state ESI-MS spectra.

Sequence	Start residue	End residue	Intensity
SLTRLQAESSAAIHATAK	63	80	8.40E+07
LQAESSAAIHATAK	67	80	1.59E+11
LQAESSAAIHATAKWTTENLAK	67	88	3.75E+07
WTTENLAK	81	88	9.17E+10
WTTENLAKTQAAQAER	81	96	4.47E+07
TQAAQAERAKAAMLSQQAAK	89	108	1.42E+08
AKAAMLSQQAAK	97	108	9.75E+07
AAMLSQQAAKAKQAK	99	113	1.65E+08
AAMLSQQAAK	99	108	2.22E+07
QAKLTQHLK	111	119	8.86E+07
LTQHLKDVVDR	114	124	2.84E+10
LTQHLKDVVDRALQNNK	114	130	1.07E+07
DVVDRALQNNK	120	130	6.58E+06
ALQNNKTRPTVIDLAHQNNQQMAAMAEFIGR	125	155	7.70E+07
TRPTVIDLAHQNNQQMAAMAEFIGR	131	155	7.03E+09
PTVIDLAHQNNQQMAAMAEFIGR	133	155	6.81E+08
RAEEAYQAALR	172	182	2.72E+07
AEEAYQAALR	173	182	4.87E+06

Supplementary Table 4. KlebC$_{1\text{-}254}$ peptides protected from trypsin digest when in complex with *Kq*TolC span residues 63-182. SDS-PAGE bands were trypsin digested and analysed by peptide mass finger printing.

Plasmid	Insert	Parent Vector
pNGH264	KlebC (Klebicin $C_{1\text{-}517}$-E9 DNase/Im9)	pET21a
pNGH317	*Kq* TolC	pET24a
pNGH318	$KlebC_{1\text{-}254}$	pET24a
pNGH319	$KlebC_{1\text{-}271}$	pET24a
pNGH320	$KlebC_{51\text{-}254}$	pET24a
pNGH349	A107C, Y177C KlebC	pET21a
pNGH350	L86C, L198C KlebC	pET21a
pNGH352	$Q204C_{1\text{-}254}$	pET24a
pNGH357	A107C, Y177C $KlebC_{1\text{-}254}$	pET24a
pNGH358	L86C, L198C $KlebC_{1\text{-}254}$	pET24a
pNGH365	M16S, V17G, L19G, KlebC	pET21a

Supplementary Table 5. Expression plasmids used in this study. All constructs were cloned such that the C-terminus contained an LE linker followed by a His_6-tag. All expression plasmids used in this study are available upon request from the corresponding author.

Primer	Sequence (from 5' to 3')
$KlebC_{1-517}$_NcoI_F	CTACGTGATGGCCATGGAGTAACGCAAAGGCGTGAAAGACTATGGGC
$KlebC_{1-517}$_NcoI_R	GCGTTACTCCATGGCCATCACGTAGATCGGTTCTACGCCGG
NdeI_*Kq*TolC_F	AAAAAACATATGAAGAAATTGCTCCCCATTCTTATCGGCC
XhoI_*Kq*TolC_R	AAAAAACTCGAGCTGACGGAACGGATTGCTGCCG
NdeI_$KlebC_1$_F	GATATACATATGGCCGACAACCAGCCGG
NdeI_$KlebC_{51}$_F	ATTATTCATATGAGCGGTAGTCTCCAGGCCG
XhoI_$KlebC_{254}$_R	CAAGGTCTCGAGAGGGGTGTCTTTGATCCCTGC
XhoI_$KlebC_{271}$_R	CAGCCGCTCGAGTGGGGTAAGGAACAGTGTTGTCGCCGC
L86C_KlebC_F	CCGCAAAATGGACCACGGAAAACTGCGCCAAGACACAGGCCGC
L86C_KlebC_R	GCGGCCTGTGTCTTGGCGCAGTTTTCCGTGGTCCATTTTGCGG
A107C_KlebC_F	GCTGAGCCAACAAGCGTGCAAAGCTAAACAAGCGAAACTGACCC
A107C_KlebC_R	GGGTCAGTTTCGCTTGTTTAGCTTTGCACGCTTGTTGGCTCAGC
Y177C_KlebC_F	CCAAACGTGCTGAAGAAGCGTGCCAGGCCGCGTTGCGTGCG
Y177C_KlebC_R	CGCACGCAACGCGGCCTGGCACGCTTCTTCAGCACGTTTGG
L198C_KlebC_F	CAGGCCGAAATCGAACGCAAGTGCCAAGAAGCCCGCAAGCAGG
L198C_KlebC_R	CCTGCTTGCGGGCTTCTTGGCACTTGCGTTCGATTTCGGCCTG
Q204C_KlebC_F	GCTGCAAGAAGCCCGCAAGTGCGAAGCTGCGGCGAAAGCG
Q204C_KlebC_R	CGCTTTCGCCGCAGCTTCGCACTTGCGGGCTTCTTGCAGC
M16S,V17G,L19G_F	CGCCAGCCCCACCAGGCAGCGGCAGCGGCGGTGTGAACGAGAACGGAGAGG
M16S,V17G,L19G_R	CCTCTCCGTTCTCGTTCACACCGCCGCTGCCGCTGCCTGGTGGGGCTGGCG

Supplementary Table 6. Primers used for cloning and mutagenesis in this study.
