# Supplementary material for: Toxin import through the antibiotic efflux channel TolC
Source: Nat Commun. 2021 Jul 30;12:4625. doi: 10.1038/s41467-021-24930-y (PMC8324772; doi:10.1038/s41467-021-24930-y)
Supplement: Supplementary file 2 — Reporting Summary [file 41467_2021_24930_MOESM2_ESM.pdf]

nature research

Corresponding author(s): Colin Kleanthous

Last updated by author(s): Jun 15, 2021

Reporting Summary

Nature Research wishes to improve the reproducibility of the work that we publish. This form provides structure for consistency and transparency in reporting. For further information on Nature Research policies, see our Editorial Policies and the Editorial Policy Checklist.

Statistics

For all statistical analyses, confirm that the following items are present in the figure legend, table legend, main text, or Methods section.

n/a	Confirmed	
☐	☒	The exact sample size (n) for each experimental group/condition, given as a discrete number and unit of measurement
☐	☒	A statement on whether measurements were taken from distinct samples or whether the same sample was measured repeatedly
☒	☐	The statistical test(s) used AND whether they are one- or two-sided<br>*Only common tests should be described solely by name; describe more complex techniques in the Methods section.*
☐	☒	A description of all covariates tested
☐	☒	A description of any assumptions or corrections, such as tests of normality and adjustment for multiple comparisons
☐	☒	A full description of the statistical parameters including central tendency (e.g. means) or other basic estimates (e.g. regression coefficient) AND variation (e.g. standard deviation) or associated estimates of uncertainty (e.g. confidence intervals)
☒	☐	For null hypothesis testing, the test statistic (e.g. F, t, r) with confidence intervals, effect sizes, degrees of freedom and P value noted<br>*Give P values as exact values whenever suitable.*
☒	☐	For Bayesian analysis, information on the choice of priors and Markov chain Monte Carlo settings
☒	☐	For hierarchical and complex designs, identification of the appropriate level for tests and full reporting of outcomes
☒	☐	Estimates of effect sizes (e.g. Cohen's d, Pearson's r), indicating how they were calculated

Our web collection on statistics for biologists contains articles on many of the points above.

Software and code

Policy information about availability of computer code

Data collection	Generic Data Acquisition as implemented at Diamond<br>GeneSys 1.6.6.0<br>FluorEssence 3.5<br>Unicorn 7.0<br>Malvern iTC200 1.26.4<br>Pro-Data SX (Applied Photophysics)
Data analysis	DIALS 1.14.13<br>SHELXD 2013/2<br>PHASER 2.8.2<br>BUCANNEER 1.6.5<br>COOT 0.8.9.2 and 0.9.4.1<br>BUSTER 2.10.3<br>SIMPLE 3.0.0-beta<br>Motion Corr 2<br>CryoSparc 2.12<br>ctffind 4<br>Relion-3.0<br>Relion-3.1<br>LocScale v0.1<br>Phenix 1.19.1-4122<br>Chimera 1.15

Chimerax 1.1.1
Pymol 2.3.1
MaxQuant 1.6.3.4
Origin 7.0
OriginPro 9.5.1.195
Pro-Data Viewer (Applied Photophysics)
Excel for Microsoft 365

For manuscripts utilizing custom algorithms or software that are central to the research but not yet described in published literature, software must be made available to editors and reviewers. We strongly encourage code deposition in a community repository (e.g. GitHub). See the Nature Research guidelines for submitting code & software for further information.

Data

Policy information about availability of data

All manuscripts must include a data availability statement. This statement should provide the following information, where applicable:

- Accession codes, unique identifiers, or web links for publicly available datasets
- A list of figures that have associated raw data
- A description of any restrictions on data availability

The data supporting the findings of the study are available in the article and its Supplementary Information. The source data underlying Figs. 1, 3, 4 and Supplementary Figs. 2, 3 and 4 are provided as a Source Data file with this paper. The atomic coordinates and structure factors of KlebC51-254, KqTolC and the KqTolC·KlebC1-254 complex have been submitted to the protein structure data bank (PDB ID codes 7NNA [https://doi.org/10.2210/pdb7NNA/pdb], 7NG9 [https://doi.org/10.2210/pdb7NG9/pdb] and 7NG8 [https://doi.org/10.2210/pdb7NG8/pdb] respectively). Electron microscopy density maps have been submitted to the EMDB with accession codes EMD-12310 and EMD-12309 for KqTolC and KqTolC·KlebC1-254 respectively.

Field-specific reporting

Please select the one below that is the best fit for your research. If you are not sure, read the appropriate sections before making your selection.

☒ Life sciences ☐ Behavioural & social sciences ☐ Ecological, evolutionary & environmental sciences

For a reference copy of the document with all sections, see nature.com/documents/nr-reporting-summary-flat.pdf

Life sciences study design

All studies must disclose on these points even when the disclosure is negative.

Sample size	For ITC duplicate titrations were performed with between 16 and 25 injections per titration to ensure adequate data coverage whilst maintaining a suficient signal to noise ratio. Stopped-flow experiments were performed in duplicate or triplicate with each experiment consisting of between 5 and 7 concentrations, such that association rate constants were derived from at least 14 data aquisitions.
Data exclusions	Following cryo-EM data collection a small number of micrographs were excluded due to poor ice and failed CTF estimate.
Replication	All experiments were performed in at least duplicate and all attempts at replication were successful.
Randomization	Randomization was not relevant to this study
Blinding	The investigators were not blinded to the sample identity as this was not relevant to the structural biology aspects of the study and was not deemed necessary for in vitro characterisation.

Reporting for specific materials, systems and methods

We require information from authors about some types of materials, experimental systems and methods used in many studies. Here, indicate whether each material, system or method listed is relevant to your study. If you are not sure if a list item applies to your research, read the appropriate section before selecting a response.

Materials & experimental systems

n/a	Involved in the study	
☒	☐	Antibodies
☒	☐	Eukaryotic cell lines
☒	☐	Palaeontology and archaeology
☒	☐	Animals and other organisms
☒	☐	Human research participants
☒	☐	Clinical data
☒	☐	Dual use research of concern

Methods

n/a	Involved in the study	
☒	☐	ChIP-seq
☒	☐	Flow cytometry
☒	☐	MRI-based neuroimaging
